# A Quantitative Model of the GIRK1/2 Channel Reveals That Its Basal and Evoked Activities Are Controlled by Unequal Stoichiometry of Gα and Gβγ

**DOI:** 10.1371/journal.pcbi.1004598

**Published:** 2015-11-06

**Authors:** Daniel Yakubovich, Shai Berlin, Uri Kahanovitch, Moran Rubinstein, Isabella Farhy-Tselnicker, Boaz Styr, Tal Keren-Raifman, Carmen W. Dessauer, Nathan Dascal

**Affiliations:** 1 Department of Physiology and Pharmacology and Sagol School of Neuroscience, Tel Aviv University, Tel Aviv, Israel; 2 Department of Integrative Biology and Pharmacology, University of Texas Health Science Center, Houston, Texas, United States of America; Rush University Medical Center, UNITED STATES

## Abstract

G protein-gated K^+^ channels (GIRK; Kir3), activated by Gβγ subunits derived from G_i/o_ proteins, regulate heartbeat and neuronal excitability and plasticity. Both neurotransmitter-evoked (I_evoked_) and neurotransmitter-independent basal (I_basal_) GIRK activities are physiologically important, but mechanisms of I_basal_ and its relation to I_evoked_ are unclear. We have previously shown for heterologously expressed neuronal GIRK1/2, and now show for native GIRK in hippocampal neurons, that I_basal_ and I_evoked_ are interrelated: the extent of activation by neurotransmitter (activation index, R_a_) is inversely related to I_basal_. To unveil the underlying mechanisms, we have developed a quantitative model of GIRK1/2 function. We characterized single-channel and macroscopic GIRK1/2 currents, and surface densities of GIRK1/2 and Gβγ expressed in *Xenopus* oocytes. Based on experimental results, we constructed a mathematical model of GIRK1/2 activity under steady-state conditions before and after activation by neurotransmitter. Our model accurately recapitulates I_basal_ and I_evoked_ in *Xenopus* oocytes, HEK293 cells and hippocampal neurons; correctly predicts the dose-dependent activation of GIRK1/2 by coexpressed Gβγ and fully accounts for the inverse I_basal_-R_a_ correlation. Modeling indicates that, under all conditions and at different channel expression levels, between 3 and 4 Gβγ dimers are available for each GIRK1/2 channel. In contrast, available Gα_i/o_ decreases from ~2 to less than one Gα per channel as GIRK1/2's density increases. The persistent Gβγ/channel (but not Gα/channel) ratio support a strong association of GIRK1/2 with Gβγ, consistent with recruitment to the cell surface of Gβγ, but not Gα, by GIRK1/2. Our analysis suggests a maximal stoichiometry of 4 Gβγ but only 2 Gα_i/o_ per one GIRK1/2 channel. The unique, unequal association of GIRK1/2 with G protein subunits, and the cooperative nature of GIRK gating by Gβγ, underlie the complex pattern of basal and agonist-evoked activities and allow GIRK1/2 to act as a sensitive bidirectional detector of both Gβγ and Gα.

## Author Summary

Many neurotransmitters and hormones inhibit the electric activity of excitable cells (such as cardiac cells and neurons) by activating a K^+^ channel, GIRK (**G** protein-gated **I**nwardly **R**ectifying **K** ^+^ channel). GIRK channels also possess constitutive “basal” activity which contributes to regulation of neuronal and cardiac excitability and certain disorders, but the mechanism of this activity and its interrelation with the neurotransmitter-evoked activity are poorly understood. In this work we show that key features of basal and neurotransmitter-evoked activities are similar in cultured hippocampal neurons and in two model systems (mammalian HEK293 cells and *Xenopus* oocytes). Using experimental data of the neuronal GIRK1/2 channel function upon changes in GIRK and G protein concentrations, we constructed a mathematical model that quantitatively accounts for basal and evoked activity, and for the inverse correlation between the two. Our analysis suggests a novel and unexpected mechanism of interaction of GIRK1/2 with the G protein subunits, where the tetrameric GIRK channel can assemble with 4 molecules of the Gβγ subunits but only 2 molecules of Gα. GIRK is a prototypical effector of Gβγ, and the unequal stoichiometry of interaction with G protein subunits may have general implications for G protein signaling.

## Introduction

G proteins and the linked G protein-coupled receptors (GPCRs) are prominent regulators of excitability, which activate or inhibit ion channels by a variety of mechanisms [1]. This paper focuses on the quantitative analysis of the classical GPCR-initiated signaling cascade that culminates in the activation of GIRK channels (G protein-gated K^+^ channel; Kir3). GIRKs are important transducers of inhibitory neurotransmitter effects in heart and brain. They regulate heartbeat, neuronal excitability and plasticity, analgesia, alcohol and drug effects, and are implicated in a number of disorders such as epilepsy, Down syndrome, bipolar disorder, atrial fibrillation and primary aldosteronism [2,3,4,5,6]. GIRK is also the first-discovered effector of Gβγ [7] and a prototypical model of membrane-delimited G protein signaling. In the now classical scheme, the agonist-bound GPCR catalyzes GDP/GTP exchange at Gα and the separation of Gα_i/o_^GTP^ from Gβγ; Gβγ directly binds to GIRK and triggers channel opening [8,9,10,11].

Mammalian GIRKs are usually heterotetramers of GIRK1 with one of the other subunits (GIRK2, GIRK3 and GIRK4). GIRK1/2 is predominant in mammalian brain, but heterotetrameric GIRK1/3, GIRK2/3 and homotetrameric GIRK2 are also abundant in certain brain regions [2]. A GIRK channel is activated by direct binding of up to 4 molecules of Gβγ, but partial activation is achieved by binding of 1–3 Gβγ molecules [12,13,14,15,16]. NMR studies [17], crystal structure [18] and docking models [19] of GIRK-Gβγ complexes have confirmed the 4:1 Gβγ:GIRK stoichiometry, showing binding of one Gβγ to each interface between adjacent GIRK subunits. Further, a strong association of GIRKs with Gβγ has been suggested by co-immunoprecipitation and Förster/Bioluminescence Resonance Energy Transfer (FRET/BRET, respectively) [20,21,22,23,24]. In support, in *Xenopus* oocytes, GIRK1-containing channels recruit Gβγ to the plasma membrane (PM) [25]. GIRK also binds Gα_i/o_ subunits which regulate the channel's basal activity, specificity and kinetics of signaling [26,27,28,29,30,31,32,33,34], but the mechanisms are poorly understood. No FRET between GIRK subunits and Gα_i/o_ could be detected in the PM [24,35,36]; GIRK1 does not recruit Gα_i_ to the PM [25] and binds Gα_i/o_ *in vitro* less strongly than Gβγ [36]. The stoichiometry of Gα-GIRK interaction is unknown.

Traditionally, GIRKs have been regarded as inhibitory devices operated exclusively by inhibitory neurotransmitters which elicit the GIRK's evoked response (I_evoked_). However, recent studies revealed that neuronal GIRKs also have a substantial basal activity, I_basal_ [37,38,39]. GIRK's basal activity and the balance between I_basal_ and I_evoked_ are important determinants of neuronal excitability [39,40], bistability of neuronal networks [41], neuronal plasticity [42,43,44], dendritic integration [45], atrial arrhythmia and remodeling [46], and have recently been proposed to be related to effects of Li^+^, a drug used in the treatment of bipolar disorder [47]. Thus, changes in I_basal_ and its relation to I_evoked_ are physiologically relevant and need to be understood.

The molecular mechanisms of I_basal_ and I_evoked_ have been extensively studied in heterologous model systems, mainly *Xenopus* oocytes and human embryonic kidney (HEK) cells (e.g. [48,49]). We discovered that, for the neuronal GIRK1/2, I_basal_ and I_evoked_ are coupled. Incremental expression of GIRK1/2 in *Xenopus* oocytes revealed an inverse correlation between I_basal_ and the extent of activation by transmitter. The higher I_basal_, the smaller the index of activation by the transmitter (R_a_) and by coexpressed Gβγ (R_βγ_) [30]. The I_basal_-I_evoked_ coupling was regulated by Gα_i_: coexpression of Gα_i3_ reduced I_basal_, increased agonist- and Gβγ-induced GIRK currents (a phenomenon we dubbed "priming" by Gα_i/o_”), and eliminated the inverse correlation between I_basal_ and R_a_ [30,31,50,51]. These findings compelled an unusual explanation of the underlying mechanism. We proposed that Gβγ available for GIRK regulation is in excess over Gα_i/o_, thus the high I_basal_ of GIRK1/2. We suggested that the magnitude of I_basal_ and its relation to I_evoked_ are crucially regulated by the availability of Gα_i/o_ [30,31,50]. Here we demonstrate that cultured hippocampal neurons show the same inverse relation between I_basal_ and R_a_ as previously found in oocytes and HEK cells. This prompted us to further use these heterologous systems to address the coupling between GIRK's basal and evoked activity.

In the present work we have developed a quantitative model for I_basal_ and I_evoked_ of GIRK1/2, which uses experimentally determined micro- and macroscopic parameters of GIRK1/2 currents and surface densities and accurately simulates and predicts macroscopic GIRK1/2 currents under a variety of conditions. Furthermore, modeling allowed to assess the apparent molar ratios of Gα and Gβγ available for GIRK, which we term “functional stoichiometry”. Our analysis reveals that, in *Xenopus* oocytes, HEK cells, and hippocampal neurons, 3 to 4 Gβγ molecules are available for the activation of GIRK1/2 channel over a wide range of surface densities of the channel, even when no exogenous Gβγ is coexpressed with GIRK. Calculations in *Xenopus* oocytes suggest a substantial increase in total concentration of Gβγ in the PM when large amounts of GIRK1/2 are expressed, corroborating the proposed mechanism of recruitment of Gβγ by GIRK1 to the PM [25]. In contrast, modeling shows that at most two Gα molecules are available for channel’s activation, even after overexpression of Gα_i3_. Furthermore, the Gα/GIRK ratio decreases with increasing channel density. The unequal and variable stoichiometry of GIRK1/2-associated Gα and Gβγ qualitatively and quantitatively explains the inverse R_a_-I_basal_ relation. Our results indicate a significant extent of association between GIRK1/2 and Gβγ, and support the notion that Gα is a non-obligatory partner in the GIRK-G protein signaling complex [50], but Gα^GDP^ plays a crucial role in regulating basal activity and, consequently, the magnitude of agonist response.

## Results

### Extent of agonist activation is inversely related to I_basal_ in hippocampal neurons

**Fig 1:**
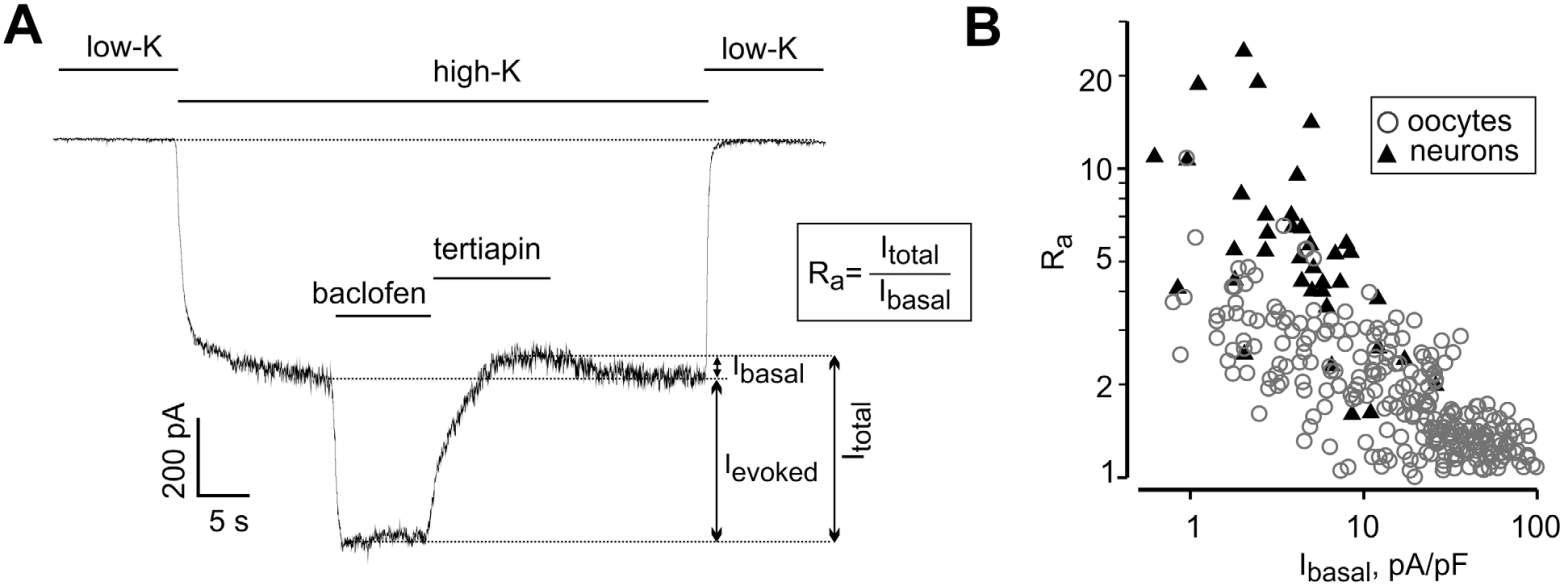
Basal and agonist-evoked GIRK currents in neurons and oocytes are inversely related. (**A**) A representative whole-recording of GIRK current in a neuron. Switching from low-K^+^ extracellular solution to a high-K^+^ solution led to the development of a large inward current probably carried by several ion channel types. Addition of baclofen elicited I_evoked_. Arrows show the amplitudes of I_basal_, I_evoked_ and I_total_. Extent of activation, R_a_, is defined as I_total_/I_basal_. (**B**) Inverse correlation between I_basal_ and R_a_ in oocytes and neurons. To allow direct comparison of I_basal_ in oocytes and neurons, currents in neurons were corrected for the 10 mV difference in holding potential, which was -70 mV in neurons and -80 mV in oocytes (see Methods). The correlation between R_a_ and I_basal_ was highly significant, p = 0.000000028 (neurons; n = 60; correlation coefficient = -0.633) and p = 0.0000002 (oocytes; n = 272; correlation coefficient = -0.728) by Spearman correlation test.

First, we wanted to characterize the relation between GIRK’s I_basal_ and I_evoked_ in hippocampal neurons, known to preferentially express GIRK1/2 [2]. I_basal_ and I_evoked_ were measured using standard experimental paradigms ([39,47]; Fig 1A). Baclofen was used to activate the endogenous GABA_B_ receptor and to generate I_evoked_ [52]. Net GIRK’s I_basal_ was revealed as shown in Fig 1A (see also S1 Fig), by adding 100–120 nM tertiapin-Q (TPNQ), which selectively blocks >90% of GIRK currents in hippocampal neurons [39,43,53].

To characterize the relation between I_basal_ and I_evoked_, we utilized the activation index R_a,_ defined as I_total_/I_basal_ (where I_total_ is the total GIRK current; see Fig 1 and S1 Fig) [51]. GIRK currents of cultured hippocampal neurons showed considerable variability: I_basal_, 0.2–26 pA/pF, I_evoked_, 1–65 pA/pF (n = 65). Strikingly, there was a strong inverse correlation between R_a_ and I_basal_ (Fig 1B, closed triangles), which was similar to that observed in oocytes expressing GIRK1/2 (open circles). The strength of the correlation indicates that it may be driven by a distinct molecular mechanism of potential physiological importance. The similarity of this distinctive phenomenon in hippocampal neurons and GIRK1/2-expressing oocytes encouraged us to further investigate it in the *Xenopus* oocyte expression system. The oocyte is particularly suitable for accurate control of protein expression (by titrating the injected RNA) and for current measurements, which are essential for quantitative modeling of I_basal_ and I_evoked_ of GIRK1/2.

### Modeling the steady-state gating of GIRK1/2 by Gβγ

Gβγ is well-established as the main gating agent for GIRK’s I_evoked_ [8,9]. This is also true for I_basal_ of heterologously expressed GIRK1/2, which is suppressed by up to 80–90% by the expression of Gβγ-binding proteins such as C-terminus of β-adrenergic kinase, phosducin or Gα, both in *Xenopus* oocytes [30,31,50] and HEK293 cells [28]. In this work, we did not manipulate cellular levels of phosphatidylinositol diphosphate (PIP_2_), and used healthy cells which always showed robust GIRK currents, indicating levels of PIP_2_ sufficient for channel activation [54]. Thus, under the conditions used in this work, Gβγ was the main gating factor determining the steady-state macroscopic GIRK current (I).

In a general form, I is described [1] by:

$$\text{I }={\text{ I}}_{\text{single}}\cdot {\text{P}}_{\text{o}}\cdot \text{N,}$$ (1)

where I_single_ is the single-channel current, N is the number of functional channels in the PM, and P_o_ is the channel’s open probability. In a heterologous expression system, the channel’s surface density (N/S, where S is the surface area of the cell) can be experimentally manipulated and measured. I_single_ of GIRK channels is an activation-independent parameter; P_o_ is the gating parameter that changes as a function of the concentration of Gβγ available for GIRK activation by agonist or added Gβγ [9,11].

**Fig 2:**
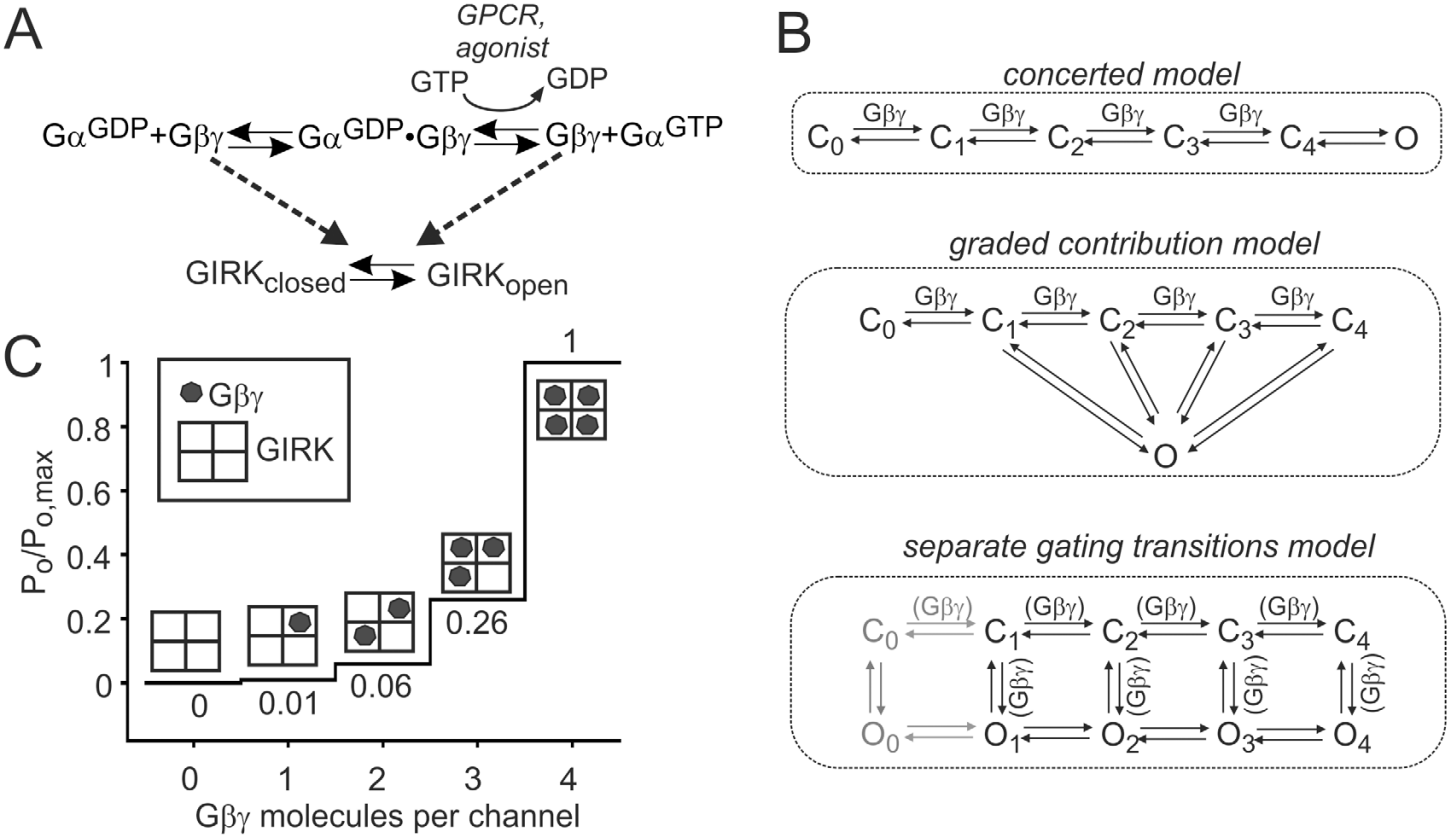
Gating of GIRK1/2 by Gβγ. (**A**) Sources of Gβγ for GIRK activation. Gα^GDP^●Gβγ is the undissociated G protein heterotrimer. Note that, in isolated Xenopus oocytes or HEK cells, in the absence of added agonist the right, GPCR-dependent branch of the reaction of Fig 2A does not significantly contribute to I_basal_, because there are no known Gα_i/o_-coupled GPCRs or ambient agonists that can "basally" activate the GTPase cycle (discussed in [51]). (**B**) The schemes of “concerted”, “graded contribution” and “separate gating transitions” models of channel activation. (**C**) Graded contribution of the four Gβγ-occupied GIRK states to P_o_. Fractional P_o_ for each state was calculated by normalizing published P_o_ values [13] of each of the four modes (corresponding to 1–4 Gβγ occupied state) to P_o,max_ (corresponding to 4 Gβγ occupied channel). Almost identical values have been obtained from fractional activation ratios for engineered GIRK channels having 1 to 4 Gβγ binding sites [14].

We start the development of the model by considering how Gβγ, available for activation of GIRK, can be derived from heterotrimeric Gαβγ (Fig 2A). In the absence of GPCR-activated G protein cycle, a small fraction of G proteins dissociates into free Gα^GDP^ and Gβγ due to finite affinity of their interaction [55,56] (the left branch of the reaction in Fig 2A). This free Gβγ can contribute to I_basal_ [57,58]. Addition of agonist activates the GPCR and promotes GDP-GTP exchange at Gα and full or partial separation of Gα^GTP^ from Gβγ (the right branch of the reaction in Fig 2A; [59,60,61]). In our experiments in *Xenopus* oocytes and HEK293 cells, we coexpressed the muscarinic receptor 2 (m2R) which couples to G_i/o_, and used acetylcholine (ACh) at supramaximal doses [62], 2–10 μM, in order to achieve a complete separation/rearrangement between Gα^GTP^ and Gβγ [63,64].

There are two existing models of GIRK gating by Gβγ. The allosteric kinetic model of Kurachi and colleagues, developed for cardiac GIRK1/4 [65,66,67], adequately describes the kinetics and magnitude of agonist- and GTPγS-evoked currents and the effect of RGS proteins. However, this model does not address I_basal_, does not include an explicit Gβγ binding step (Gβγ acts catalytically) and assumes very high surface densities of G proteins, in the order of 56 mM, which are incompatible with our measurements or those of others (see below and Discussion). It would be difficult to adjust this model for our purposes and to adequately describe I_basal_, or to implement the Gβγ recruitment phenomenon.

The second model, termed here “concerted model”, was previously developed by us to describe the G protein-dependent activation of GIRK by Na^+^ (Fig 2B) [58]. The model included a description of I_basal_ and an explicit Gβγ-GIRK binding step, but not the Gα^GTP^-Gβγ dissociation step or I_evoked_. Further, it assumed opening of the channel only when all four Gβγ binding sites are occupied (Fig 2B), which does not concur with the experimental findings that suggest a graded contribution of each bound Gβγ molecule [13,14,68].

Therefore, in the present work, we have developed a “graded contribution” model (Fig 2B and 2C), where each Gβγ-occupied state can contribute to channel opening and thus to P_o_ [13,14,68]. To date it is not known whether Gβγ binding to GIRK is truly cooperative (i.e. whether Gβγ occupancy at one binding site increases the affinity of Gβγ binding at another site). Therefore, for simplicity, in the graded contribution model we assume that Gβγ binding to GIRK is sequential and the affinity of each Gβγ-binding site is independent of the occupancy of other sites. However, overall the process of gating is cooperative, since occupancy of each additional Gβγ-binding site increases P_o_ in a more-than-additive manner (Fig 2C). The relative contributions of each Gβγ-occupied channel state to P_o_ are adopted from published data for the homologous GIRK1/4 channel [13,14]. Thus, one bound Gβγ causes channel opening with a P_o_ which is 1% of the maximal P_o_, P_o,max_; two Gβγ give 6%, three Gβγ 26%, and four Gβγ 100% of P_o,max_ (Fig 2C). This approach is applicable only to steady-state calculations of macroscopic currents since it omits the kinetic details, but it allows to bypass the need to determine (or assume) a large number of unknown parameters: state-dependent changes in channel’s affinity to Gβγ, rates of closed-open transitions from different Gβγ-bound states, and the contributions of several potential open states. Once the channel achieved a state with n Gβγ bound, its fractional P_o_ is known and does not depend on the pathway by which the channel opens. For simplicity, in calculating the steady-state P_o_ for each Gβγ-occupied state, all open states (usually 2 are reported for GIRKs; [69,70,71,72]) were pooled into a single one (see Fig 2B).

We have also considered a more general model with 4 separate closed states, in which each closed subunit can open independently of other subunits and the opening is promoted by Gβγ binding, giving rise to four open states (the “separate gating transitions model”, Fig 2B). The scheme shown also describes an alternative case in which subsequent closed states C_1_-C_4_ arise from the Gβγ-free closed state C_0_, the transitions between closed states are driven by Gβγ binding, and there are 5 separate C-O transitions. In both cases, it can be shown that, utilizing the approach based on graded contributions of each Gβγ-occupied channel state to P_o_, the derivation of P_o_ converges to the same lead equation (eq 6) as for the graded contribution model (see Supplemental Discussion, S2 Text). Therefore, in this study we implemented the graded contribution model to simplify calculations. Throughout this work we also used an extended version of the concerted model, with the inclusion of the GPCR-induced dissociation of Gα^GTP^ from Gβγ, to cross-check the conclusions of the graded contribution model.

Quantitative description and modeling of signaling cascades require the evaluation of amounts, stoichiometry, and affinities of interactions of participating proteins [73,74,75,76,77]. We took an approach that rests as much as possible on experimentally determined parameters. The data necessary for simulation by the model (eqs 5–13 in Methods) are the surface density of GIRKs and G proteins in *Xenopus* oocytes, macroscopic parameters of GIRK1/2 gating (whole-cell I_basal_, I _evoked_ and the current induced by coexpression of Gβγ, I_βγ_, see S2 Fig) at different channel densities, and I_single_ and P_o,max_. Other parameters were experimentally determined in other works.

### Single-channel currents and P_o,max_ of GIRK1/2

**Fig 3:**
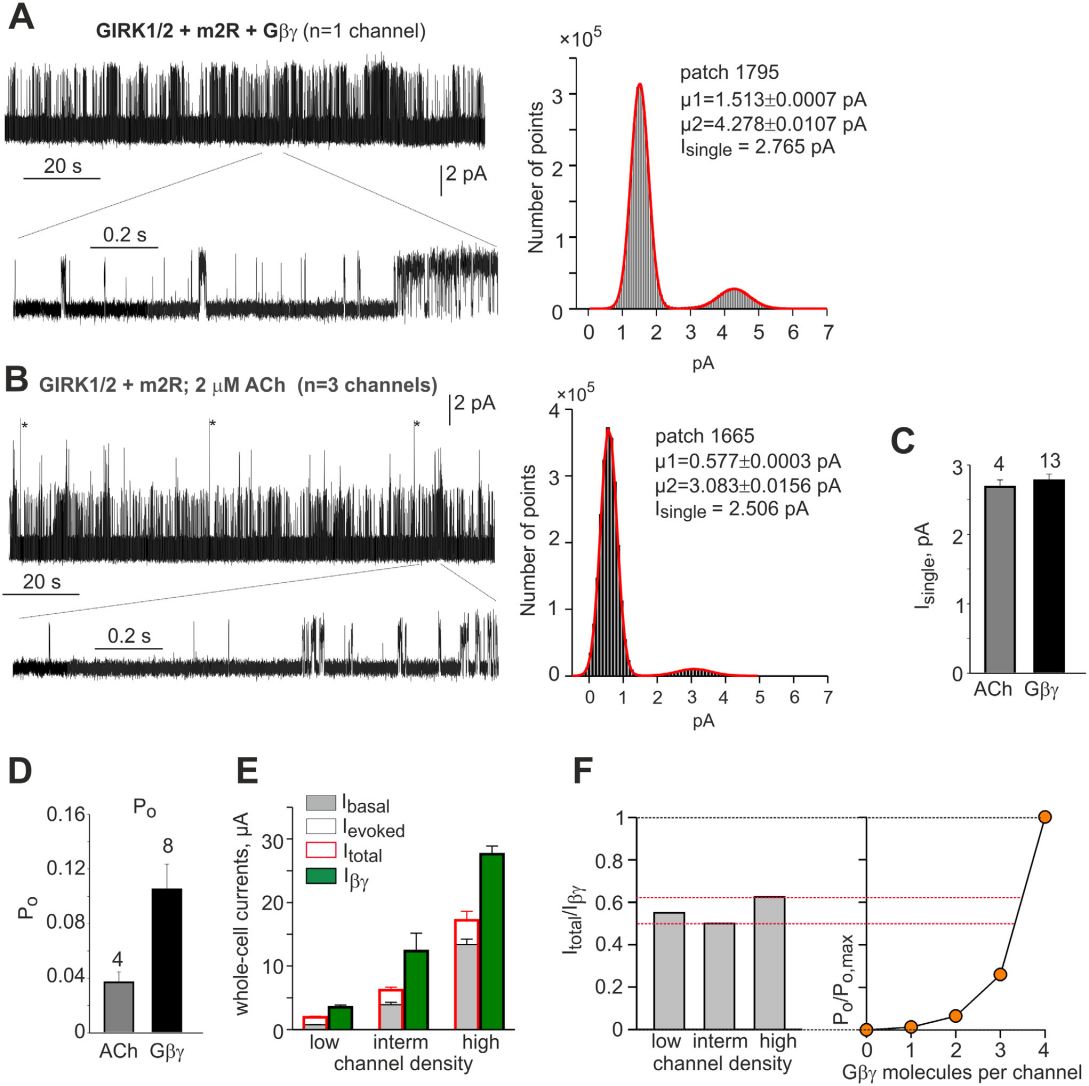
Single channel and whole-cell data reveal incomplete activation of GIRK1/2 by agonist compared to Gβγ. **(A)** Activity of GIRK1/2 in a cell-attached patch of an oocyte expressing the channel, m2R and Gβγ, without an agonist in the pipette. Right panel shows a 2 minutes segment of record, with zoom (below) on a shorter segment. The amplitude distribution histogram of the same 2 min-segment is shown on the right. Red line shows a two-component Gaussian fit. I_single_ was determined as the difference between the fitted midpoints (μ) of the GIRK current peak on the right (μ2) and the left peak which corresponds to noise (μ1). **(B)** Activity of GIRK1/2 channels in a cell-attached patch of an oocyte expressing the channel and m2R and activated by 2 μM ACh present in the patch pipette. (Asterisks denote artifacts produced by capacity discharges of patch clamp headstage). The corresponding amplitude histogram of the 2 min-segment of the record is shown on the right. In A and B, GIRK1/2 was expressed at low densities (GIRK1, 10–50 pg RNA; GIRK2, 7–17 pg RNA) whereas RNAs of m2R (1–2 ng/oocyte) and Gβγ (5:1 ng/oocyte) were chosen to produce saturating concentrations of these proteins. Inward K^+^ currents are shown as upward deflections from zero level. In the traces shown, acquisition was at 20 KHz with 5 KHz analog filter. Very similar values of I_single_ were obtained with 2 KHz filtering (not shown). **(C)** Single channel currents (left plot) are identical with either ACh or Gβγ. **(D)** P_o_ is lower with ACh than with Gβγ (p = 0.029). Bars in C and D show mean±SEM, number of patches is shown above the bars. **(E)** Summary of whole-cell GIRK1/2 currents at three expression levels (densities). See Table 1 for details. **(F)** Left panel shows the I_total_/I_βγ_ ratios at three channel densities, calculated from data of Table 1. The right panel shows the fractional open probabilities of channels occupied by 0–4 Gβγ, same as in Fig 2C but in a simple graphic form. The red dotted lines are drawn to allow direct comparison of the experimental data from the left panel with the estimates of fractional P_o_ from the right panel.

To estimate single-channel current (I_single_) and P_o_ of Gβγ- and agonist-activated GIRK1/2, we expressed the channels at low density with m2R and recorded channel activity in cell-attached patches (Fig 3A and 3B). I_single_ was determined from amplitude distribution histograms (Fig 3A and 3B; right panels). The Gaussian fits to these histograms showed one main conductance level, suggesting that subconductance states, if any, did not significantly contribute to P_o_. The average I_single_ was identical for ACh and Gβγ activation (Fig 3C), ~2.8 pA.

P_o_ was estimated from patches containing 1 to 3 channels (see Methods). When channels were activated by Gβγ expressed at a saturating dose with no agonist present, P_o_ was 0.105±0.018 (Fig 3D). The Gβγ RNA dose used (5 ng Gβ RNA, 1 ng Gγ RNA) consistently produced maximal macroscopic activation (see below, for example S8 Fig), and ACh generated negligible whole-cell I_evoked_ which was ~10% of I_basal_ (Ra = 1.1 ± 0.02, n = 14). Thus, free endogenous Gα^GTP^ produced upon activation of m2R did not substantially affect the GIRK1/2 current evoked by saturating Gβγ. We therefore conclude that the P_o_ measured in oocytes expressing saturating Gβγ is the P_o,max_ of GIRK1/2, within a possible ~10% error. In comparison, when GIRK1/2 was activated via the coexpressed m2R (no Gα or Gβγ were coexpressed) with 2–5 μM ACh in the pipette, P_o_ was 0.037±0.008, less than half of P_o,max_. (Fig 3B and 3D). (The actual P_o_ could be higher because of the desensitization observed with ACh but not with Gβγ; see Methods).

### Initial estimation of functional GIRK1/2:Gβγ stoichiometry from macroscopic currents

For further analysis and modeling of whole-cell I_basal_ and I_evoked_, we varied the surface density of GIRK1/2. The design was to obtain low, intermediate and high densities of GIRK1/2 by injecting 25, 100–200 or 1000–2000 pg RNA of each subunit per oocyte. The cells expressed 1 or 2 ng of m2R RNA which did not affect I_basal_ (S2C Fig and ref. [78]) but could always produce the maximal I_evoked_ [78]. I_evoked_ was elicited by ACh at 10 μM, a saturating dose. Under these conditions, all Gα_i/o_ should convert to Gα^GTP^, so that all available Gβγ can bind to the channel and activate it. The data are summarized in Table 1; main findings are also briefly highlighted in Fig 3E and 3F. We measured I_basal_, I_evoked_ and I_total_ in each oocyte (set 1 in Table 1, S2A Fig). In separate groups of oocytes expressing saturating Gβγ, where channel’s P_o_ reached P_o,max_, we measured I_βγ_ (set 2 in Table 1, S2B Fig).

**Table 1:** Whole-cell currents of GIRK1/2, the calculated surface density and I_βγ_/I_total_ in *Xenopus* oocytes.

Group (channel density)	ng RNA GIRK1, GIRK2	Set 1: experiments with agonist			Set 2: experiments with no agonist		calculated density (channels/μm^2^)	I_βγ_/I_total_
		I_basal_ (μA)	I_evoked_ (μA)	I_total_ (μA)	No Gβγ I_basal_ (μA)	Gβγ expressedI_βγ_ (μA)		
Low	0.025	0.73±0.065 (51)	1.19±0.09 (51)	1.92±0.14 (51)	1.06±0.13 (18)	3.49±0.37 (14)	2.74±0.29	1.82
Intermediate	0.1–02	3.9±0.36 (55)	2.3±0.2 (55)	6.2±0.45 (55)	3.77±0.55 (28)	12.34±2.82 (26)	9.7±2.2	2
High	1–2	13.36±0.87 (10)	3.84±0.81 (10)	17.2±1.42 (10)	15±0.84 (75)	27.6±1.3 (77)	21.7±1	1.6

Data are shown as mean±SEM (except I_βγ_/I_total_), number of cells is shown in parentheses. Data for each entry were collected from at least 2 independent experiments. The Table summarizes separate sets of experiments: those where I_basal_, I_evoked_ and I_total_ were measured (in each oocyte); and those where Gβγ was coexpressed and I_βγ_ was measured. In addition, in Set 2, I_basal_ was measured in each experiment in a separate group of oocytes not injected with Gβγ RNA. For the low density group in oocytes, there was ~30% difference (p = 0.017) for I_basal_ between the two sets of experiments, probably because of variability among oocyte batches. In intermediate and high density groups I_basal_ was not different (p>0.4) for both sets of experiments.

It is noteworthy that in oocytes, at all channel densities, I_βγ_ was 1.6–2 fold greater than I_total_, the total GIRK current (I_basal_ + I_evoked_) without coexpressed Gβγ (Table 1). A similar I_βγ_/I_total_ ratio of 1.66 was observed in HEK293 cells (Table 2), where all data have been pooled together (because GIRK1/2 expression levels have not been monitored). Similarly, I_βγ_/I_total_ ratio of ~2.2 for GIRK1/2 expressed in HEK cells can be estimated from the data of Wydeven et al. [79] who activated GIRK with baclofen via GABA_B_ receptors (I_evoked_ ~40 pA/pF, I_βγ_ ~ 90 pA/pF). The inverse value, I_total_/I_βγ_, ranged 0.5–0.62 at different channel densities (Fig 3F, left panel). Since GIRK1/2 was maximally activated by the doses of Gβγ used in these experiments, I_total_/I_βγ_ is equal to the fraction of maximal activation after GPCR activation, P_o_/P_o,max_. Note that the single-channel data (Fig 3D) show less than 40% activation with saturating ACh (although, as noted above, this is probably an underestimate because of desensitization). In all, in oocytes and HEK cells, when the channel is activated by an agonist, only 40–60% of maximal P_o_ is achieved.

**Table 2:** Whole-cell currents of GIRK1/2 and I_βγ_/I_total_ in HEK293 cells.

Set 1: experiments with agonist			Set 2: Gβγ expression	I_βγ_/I_total_
I_basal_ (pA/pF)	I_evoked_ (pA/pF)	I_total_ (pA/pF)	I_βγ_ (pA/pF)	
19.1±4.4 (25)	30.6±6.7 (25)	49.7±10.5 (25)	82.6±25.6 (6)	1.66

Data are shown as mean±SEM (except I_βγ_/I_total_), number of cells is shown in parentheses. Data for each entry were collected from at least 2 independent experiments, except I_βγ_ which was measured in one experiment. Raw data of Set 1 were reported in [51].

From the data of Fig 3, one can approximately estimate the amount of Gβγ molecules that are bound to the channel after maximal activation by agonist. This is done by comparing between measured values of I_total_/I_βγ_ (Fig 3F, left panel) and the expected P_o_/P_o,max_ [13,14] from Fig 2C. To facilitate the comparison, we have redrawn the plot of Fig 2C in a simple graphic form (Fig 3F, right panel), and projected the values of I_total_/I_βγ_ onto the P_o_/P_o,max_ plot (red dashed lines). For 3 bound Gβγ, the expected P_o_/P_o,max_ is 0.26, and for 4 Gβγ it is 1. Thus, with I_total_/I_βγ_ of 0.4–0.6, we estimate that, even without coexpression of Gβγ, between 3 and 4 Gβγ are available for activation of a single GIRK channel at all channel densities.

### Expression-dependent changes in surface levels of GIRK1/2

For saturating Gβγ, Eq 1 for I_βγ_ takes the form:

$${\text{I}}_{\beta \gamma }={\text{ I}}_{\text{single}}\cdot {\text{P}}_{\text{o,max}}\cdot \text{N}.$$ (2)

From here, we calculated the total number of functional channels in the PM (N) and the corresponding channel density per μm^2^ of the PM. As shown in Table 1, our "low", "intermediate" and "high" expression levels correspond to approximately 2.7, 9.7 and 21.7 channels/μm^2^, respectively. In the following, the data of Table 1 served as the basis for testing the predictions of the model and for calculating Gα and Gβγ available for channel activation.

### Surface levels of GIRK1/2 are confirmed by quantitative immunochemistry

**Fig 4:**
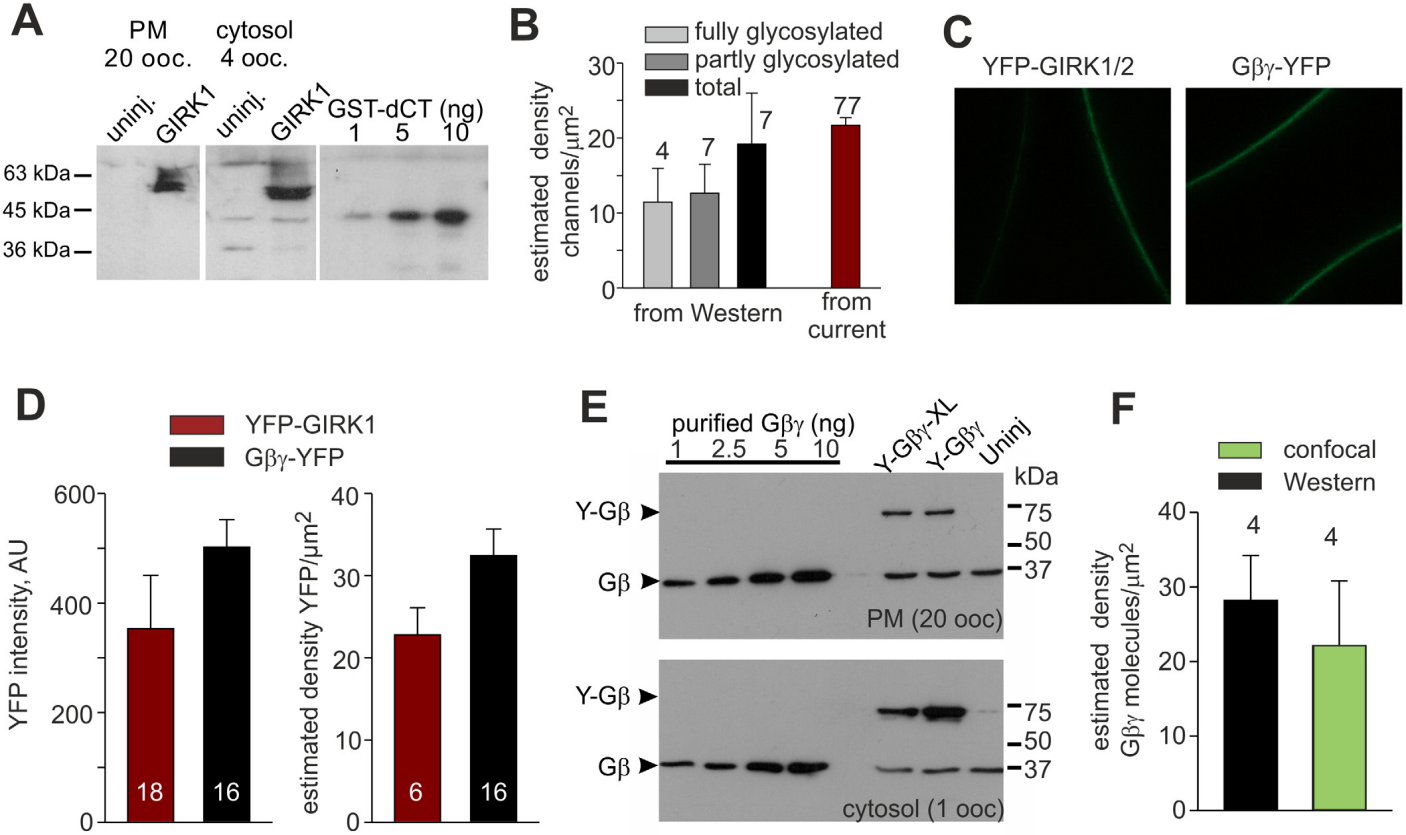
Measuring the surface density of GIRK1/2 and Gβγ in *Xenopus* oocytes. **(A)** Immunochemical estimation of the amount of GIRK1 in manually separated plasma membranes of *Xenopus* oocytes injected with 1 or 2 ng of GIRK RNA. Shown is a Western blot of 20 manually separated plasma membranes and 4 cytosols, and variable known amounts of the GST-fused distal C-terminus of GIRK1 (the antibody's epitope) used for calibration of the antibody-produced signal. There was a non-specific band at ~75 KDa in cytosols but not PM of uninjected oocytes (“uninj”). **(B)** Summary of quantitative analysis of GIRK1 in PM from Western blots of 7 separate experiments. The fully glycosylated band was observed in 4 out of 7 blots. Molar amounts of protein and PM densities from Western blots were calculated as detailed in Methods. The dark red bar is the GIRK1/2 surface density in the high-density group estimated from I_βγ_ (see Table 1), shown for comparison. **(C)** Examples of confocal images of oocytes expressing YFP-GIRK1/2 (5 ng RNA) and Gβγ-YFP (5 ng RNA). **(D)** Estimating YFP molecules density in PM using YFP-GIRK1/2 as molecular ruler. A representative experiment is shown. The left plot shows the measured intensities of YFP-GIRK1/2 and YFP-Gβ coexpressed with wt Gγ in a separate group of oocytes (5:1 ng RNA). The right plot shows the PM densities of YFP in the YFP-GIRK1/2 oocytes, calculated as follows: I_βγ_ was 14.5±2.1 μA (n = 6), corresponding to 11.4±1.6 channels/μm^2^, or 22.8±3.3 YFP molecules/μm^2^. The density of YFP in the YFP-Gβγ expressing oocytes was calculated based on relative intensities from the left plot. **(E)** Estimating the amount of endogenous Gβ and expressed YFP-Gβ or YFP-Gβ-XL (5 ng RNA) coexpressed with wt-Gγ, in manually separated plasma membranes. Protocol was similar to Fig 4A; wt purified recombinant Gβγ was used for calibration. In parallel to biochemical measurements, we also measured GIRK currents and YFP intensity in 5–15 oocytes expressing either YFP-GIRK1/2-Gβγ or YFP-Gβγ, as explained in D. **(F)** Summary of YFP-Gβγ surface density measurements in 4 experiments by the two methods, quantitative Westerns and confocal imaging with YFP-GIRK1/2 as the molecular ruler.

To obtain an independent estimate of the density of GIRK1/2 in the PM, we used quantitative immunochemistry of GIRK1 in cytosol-free, manually separated plasma membranes of *Xenopus* oocytes (Fig 4A and 4B) [80,81]. GIRK1 was coexpressed with GIRK2 at high density, and GIRK1 was probed with a C-terminally directed antibody. Western blots of manually separated PM and of the rest of the cells without the nucleus ("cytosol") showed the presence of two forms of GIRK1. A double band of about 55–58 KDa was always observed, and an additional higher diffuse band was seen in four out of seven experiments (Fig 4A and 4B). These bands correspond to partly and fully glycosylated channels, respectively [82,83]. Three oocyte batches showed only partly glycosylated bands in the PM. Since oocytes injected with 2 ng RNA always had large GIRK1/2 currents, it is likely that both partly and fully glycosylated channels are functional at the PM, in agreement with [83]. Notably, the main fraction of the channel was found in the cytosolic fraction (most likely endoplasmic reticulum and Golgi), largely in a partly-glycosylated form (Fig 4A). This is not unexpected, because in *Xenopus* oocytes the PM constitutes only a very small fraction of the cell's total mass [81].

Next, molar amounts of GIRK in the PM fraction were calculated, taking into account the presence of two GIRK1 subunits in each channel. Calibration of antibody-produced signal was done with known amounts of the GST-fused distal C-terminus of GIRK1 which contains the epitope for the antibody. Note that this method yields channel levels in concentration units (e.g. mole/L). Both GIRK and Gβγ are associated with the PM (Gβγ is membrane-anchored by a lipid moiety [84]), and the interaction between GIRK’s cytosolic domain and Gβγ takes place within the submembrane space. Therefore, to compare data with GIRK1/2 surface densities obtained by channel counting from currents (Table 1), and for further modeling, we have converted two-dimensional protein densities to protein concentrations within the interaction space as previously described (e.g. [58,85,86,87]; see Supplemental Discussion, S2 Text), according to

C = N/(W·S·A), (3)

where C is the concentration of protein in the submembrane space, N is the number of protein molecules in the membrane, S is a membrane area, A is Avogadro number and W is the width of the interaction space. For calculations, we used S_oocyte_ = 2×10^7^ μm^2^ (deduced from an oocyte's capacitance of 200 nF [88] and specific capacitance of plasma membrane of 1 μF/cm^2^), and W was assumed to be 10 nm. The latter roughly corresponds to the molecular size of the complex of Gβγ and the cytosolic part of GIRK [18]. The influence of this parameter on the conclusions of the model was tested later (see below, S6 Fig, panels D and E). Consequently, the standard conversion factor between channel density (number of channels/μm^2^) and channel concentration (nM) is 1 channel/μm^2^ = 166 nM.

Conversion of channel concentrations determined in Fig 4 into surface densities using Eq 3 gave ~12–14 channels/μm^2^ for both partly and fully glycosylated channels in the PM, and the average total amount of GIRK1/2 in PM (with partly + fully glycosylated GIRK1) was 19.1±6.8 channels/μm^2^ (Fig 4B). This is in good agreement with the independent assessment of ~22 channels/μm^2^ obtained from measurements of I_βγ_ for the high GIRK1/2 expression group (Table 1). To note, the latter was calculated using Eq 2 with P_o,max_ measured at low channel densities. If P_o,max_ were different at high GIRK1/2 expression levels, the densities calculated from I_βγ_ and immunochemistry would not match. The close correspondence between the two independent approaches indicates that P_o,max_ is preserved at the high expression level.

We conclude that the total surface density of GIRK1/2 channels in the PM can be satisfactorily estimated from whole-cell currents (Eq 2, Table 1). Such measurements are calibration-independent and accurate [1], and therefore GIRK1/2 can be used as a “molecular ruler”. In this procedure, the fluorescently labeled GIRK1/2, with its surface density calculated from I_βγ_, will serve as a reference for estimating PM densities of other fluorescently labeled proteins. To use GIRK1/2 as a molecular ruler, we expressed YFP-GIRK1 (GIRK1 with Yellow Fluorescent Protein (YFP) fused to the N-terminus). Single channel analysis of Gβγ-activated YFP-GIRK1 coexpressed with GIRK2, YFP-GIRK1/2, showed the same P_o,max_ and I_single_ as in wild-type GIRK1/2 (S3 Fig), allowing the use of this construct for calibration purposes. To obtain high current levels of YFP-GIRK1/2 we usually had to inject 5 ng/oocyte of channel’s RNA.

### Surface levels of Gβγ

To estimate the expression levels of Gβγ using YFP-GIRK1/2 as molecular ruler, we expressed YFP-GIRK1/2 and, in separate oocytes of the same batch, Gβγ in which either Gβ or Gγ was labeled with YFP. Expression of YFP was monitored from fluorescence intensity in the PM (Fig 4C). In addition, I_βγ_ was measured and the surface density of YFP-GIRK1/2 was calculated. The amount of YFP molecules per μm^2^ was calculated assuming a 2:2 GIRK1:GIRK2 stoichiometry in a heterotetramer [89] (See also Supplemental Discussion, S2 Text). The surface density of Gβγ-YFP was then calculated based on intensity ratios of YFP-GIRK1 and Gβγ-YFP. A typical experiment is shown and explained in Fig 4D.

To validate the estimates of Gβγ expression, in four experiments as in Fig 4D we also measured the levels of Gβ-YFP (coexpressed with unlabeled Gγ) by quantitative immunochemistry in manually separated plasma membranes. We used purified Gβγ to calibrate the signal produced by the Gβ antibody (Fig 4E). We also constructed and expressed an YFP-fused construct corresponding to *Xenopus laevis* Gβ1, YFP-Gβ-XL (see Methods). Western blots showed a prominent ~36 KDa band of the endogenous Gβ, and ~70 KDa bands corresponding to the expressed YFP-Gβ or YFP-Gβ-XL (Fig 4E). The surface density of the expressed YFP-Gβγ assessed by the quantitative immunochemical method was 28±6 molecules/μm^2^, close to the estimate of 22.1±8.7 molecules/μm^2^ obtained in the same experiments from measurements of fluorescence using YFP-GIRK1 as "molecular ruler" (Fig 4F; 53 oocytes, n = 4 experiments; P = 0.295). These results demonstrate the feasibility of the molecular ruler methodology and provide a good estimate of the expressed Gβγ-YFP. In several sets of experiments (see also below) we consistently found that, with 5 ng RNA of Gβγ, its surface density ranged between 20 and 30 molecules/μm^2^.

We next utilized YFP-Gβ-XL as a caliper for the endogenous oocyte's Gβ. Results of 4 experiments showed that, in Western blots, Gβ antibody used here gave similar signal with Gβ-XL as with bovine Gβ_1_ (S3 Fig, panels C, D). We then estimated the surface density of the endogenous Gβ (the 37 kDa band in Fig 4E) to be 24±4.6 molecules/μm^2^ (n = 4). We have also estimated the concentrations of total and cytosolic endogenous Gβγ from the 4 experiments of Fig 4E, assuming an oocyte’s water volume of 0.5 μl [88]. The total Gβγ concentration was 173±44 nM, the concentration of Gβγ in the cytosolic fraction was 171±44 nM.

### Estimation of functional stoichiometry of GIRK1/2, Gβγ and Gα

We define the molar amounts of proteins physically available for the function of the cascade as functional stoichiometry. It can change depending on availability of a protein, in contrast to limiting (maximal) stoichiometry which reflects the maximal molar ratios of interacting proteins. For example, if one GIRK channel can interact with at most 4 Gβγ and 4 Gα molecules, then the limiting GIRK:Gβγ:Gα stoichiometry is 1:4:4.

Having determined the P_o,max_ and surface densities of GIRK1/2 and endogenous Gβγ, we were now able to simulate macroscopic GIRK currents in oocytes and to assess the functional stoichiometry of GIRK1/2-Gα-Gβγ. We initially assumed that all of the I_basal_ in oocytes was Gβγ-dependent. The affinities of GIRK-Gβγ and Gα-Gβγ interactions were adopted from published work: K_D_ = 1.86 nM for Gα^GDP^-Gβγ binding [56], and K_D_ = 50 nM for the GIRK-Gβγ interaction, as estimated by biochemical methods [90]. Simulations were done using eqs 5–16 as explained in the Methods section. Simulated data were compared to experimental measurements of GIRK1/2 activity for the three GIRK1/2 surface density groups (Table 1 and Fig 3E). Note that GIRK densities were calculated from I_βγ_ but simulations were done for the separately measured I_basal_ and I_evoked_, avoiding circular reasoning.

We first tried to simulate the experimental data by assuming that only endogenous Gαβγ is available for the activation of GIRK1/2 (S4A Fig). However, no satisfactory description of data can be obtained under this assumption. Simulations that assumed recruitment of 3–4 Gβγ with GIRK, without Gα, gave a better approximation to the data (S4 Fig, panels B, C).

**Fig 5:**
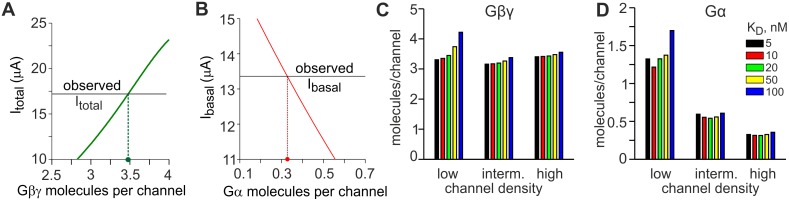
Estimation of Gβγ and Gα available for GIRK1/2 activation from macroscopic currents. (**A, B**) The method of estimation of number of Gβγ and Gα molecules per channel is exemplified for the high channel density group of Table 1. The same procedure has been applied to the low and intermediate density groups (Table 3). **(A)** Estimation of Gβγ available for channel activation utilizing I_total_. Simulated I_total_ (green line) was calculated for a range of Gβγ surface densities using eqs 5–12, and compared with the experimentally observed I_total_. **(B)** Estimation of Gα_i/o_ available for interaction with the channel. Simulated I_basal_ (red line) was calculated using eqs 5–16 for a range of Gα surface densities, using the Gβγ density calculated in (A), and compared with the experimentally observed I_basal_. (**C, D**) The estimates of Gβγ:GIRK (C) and Gα:GIRK (D) ratios are stable in a wide range of GIRK-Gβγ interaction affinities, from K_D_ = 5 nM to 100 nM. Simulations were done with the graded contribution model separately for the low-, intermediate- and high density groups from Table 1 (2.74, 9.7 and 21.7 channels/μm^2^, respectively).

Next, we turned to a more accurate assessment of functional GIRK1/2:Gβγ:Gα stoichiometry. Our model allows to calculate the amounts of Gα and Gβγ available for GIRK1/2 without any prior knowledge or assumption about the G protein concentrations in the cell, directly from experimental data. This idea is illustrated graphically in Fig 5 for the high GIRK1/2 surface density group of Table 1. The procedure consists of two steps. First, because all Gβγ available for GIRK is free to activate the channel after addition of agonist, the total GIRK-available Gβγ can be calculated from I_total_, as shown in Fig 5A. Here, the solid green line presents the simulated I_total_ for a range of GIRK-available Gβγ densities, normalized to channel density (Gβγ:GIRK ratio). The intercept of simulated I_total_ and the experimentally observed I_total_ produces the estimate of the Gβγ:GIRK ratio. The numeric calculation of Gβγ density from experimental I_total_ can also be done by substituting the channel density and I_total_ into the eqs 5–13 and 16 (see Supplemental Methods (S1 Text) for Matlab routines).

Next, the estimate of Gβγ is used to calculate the available Gα from I_basal_. I_basal_ is determined by "free" Gβγ that is not bound to Gα^GDP^ in the absence of agonist. The calculation is done by substituting channel density, I_basal_ and the estimated value of available Gβγ into the eqs 5–16 (see Supplemental Methods, S1 Text); a graphical illustration is shown in Fig 5B. The summary of calculations, made with our standard assumption of K_D_ = 50 nM for the GIRK-Gβγ interaction, is presented in Table 3 and Fig 5C and 5D. These calculations show that there are ~3–4 Gβγ, but less than 2 Gα, available for each channel. Moreover, while Gβγ:GIRK ratio remains relatively constant throughout the range of analyzed channel densities, there is a sharp decrease in Gα:GIRK ratio with the increase in channel density (Table 3; see also below and S6 Fig).

**Table 3:** Calculation of Gβγ and Gα available for channel activation in *Xenopus* oocytes (without coexpressing Gβγ). K_D_ for channel-Gβγ interaction was taken as 50 nM. For calculations with other K_D_ values, see Fig 5 and S5 Fig.

		Graded contribution model				Concerted model			
Channel density group	channels/μm^2^ (from Table 1)	total available Gα and Gβγ, molecules/μm^2^		Gβγ:GIRK and Gα:GIRK ratios		total available Gα and Gβγ, molecules/μm^2^		Gβγ:GIRK and Gα:GIRK ratios	
		Gβγ	Gα	Gβγ: GIRK	Gα: GIRK	Gβγ	Gα	Gβγ: GIRK	Gα: GIRK
Low	2.74	10.2	3.75	3.74	1.37	11.3	3.3	4.14	1.22
Intermediate	9.7	31.6	5.4	3.26	0.56	34.2	4.3	3.53	0.44
High	21.7	75.5	7.2	3.48	0.33	79.6	5.6	3.67	0.26

The estimate of K_D_ for the GIRK-Gβγ interaction varies depending on the method used, from K_D_ = 4–10 nM determined in excised patches [91,92,93] to ~50 nM in direct biochemical measurements [90]. To check for model’s stability regarding this parameter, we calculated the available Gβγ and Gα for a range of K_D_ from 5 to 100 nM. As shown in Fig 5C and 5D, the estimates of Gβγ:GIRK and Gα:GIRK ratios, and the trend in their changes as a function of channel density, remain highly stable within the examined range of K_D_. Similar conclusions were attained using the concerted model (Table 3 and S5 Fig). The latter does not involve any assumptions for fractional Gβγ contributions to total P_o_. Similarity of conclusion of the two models alleviates concerns regarding the use of values for fractional Gβγ contributions, adopted from GIRK1/4 studies, to simulate GIRK1/2.

Table 3 also shows the total Gβγ and Gα available for GIRK in PM, in molecules/μm^2^, calculated for each of the three channel densities. It is easy to see that, at intermediate and high channel densities, the endogenous Gβγ, 24 molecules/μm^2^ (that was present in the PM before the expression of GIRK) cannot account for the observed GIRK1/2 activation. In contrast, estimates of total Gα available for GIRK1/2 remain within the limits of endogenous Gα. As mentioned before, the calculations have been made without any assumption regarding the presence or amount of endogenous Gαβγ, and made no specific *a priori* assumptions regarding recruitment of Gβγ or Gα. Hence, modeling independently predicts the necessity for GIRK1/2-related increase in PM density of Gβγ, corroborating the experimentally observed recruitment of Gβγ, but not Gα, by GIRK1/2 [25].

Next, we addressed the possible contribution of intrinsic, Gβγ-independent activity to I_basal_. About 10–20% of I_basal_ of GIRK1/2 in *Xenopus* oocytes [30,31,50] and HEK293 cells [28] persists after expression of Gβγ scavengers or Gα. Whereas the residual I_basal_ may reflect incomplete Gβγ chelation, a genuine Gβγ-independent fraction of I_basal_ cannot be discarded. In the extreme case it may contribute up to 20% of GIRK1/2 I_basal_. This may account for up to 10% of P_o,max_ (because I_basal_ can reach at most half of I_βγ_, which is the indicator of P_o,max_; Tables 1 and 2).

We have therefore extended the model to include the contribution of a hypothetical intrinsic Gβγ-independent channel activity. We assume that the intrinsic basal P_o_ of a channel (P_o,intrinsic_) is an inherent, density-independent property of a single channel, best described as a fraction of P_o,max_. We thus repeated our calculations of GIRK1/2-available Gα and Gβγ assuming a P_o,intrinsic_ in the range between 1% and 10% of P_o,max_ (S6 Fig, panels A, B). For these calculations, Eq 6 (Methods) was modified in the following way:

$${\text{I=i}}_{\text{single}}\cdot \text{N}\cdot {\text{P}}_{\text{o,max}}\cdot (\phi \cdot \sum_{\text{1}}^{\text{4}}{\text{f}}_{\text{p}}{}_{\text{,x}}\cdot \phi_{\text{x}}+(1-\phi ))\text{ ,}$$ (4)

where Φ is the fraction of P_o,max_ which is Gβγ-dependent (see Eqs 6 and 7 in the Methods for definitions of other parameters). In the whole range of P_o,intrinsic_ tested, the estimation of 3–4 Gβγ per channel remained highly stable (S6 Fig). The estimate of less than 2 Gα per channel also persisted except at the highest P_o,intrinsic_ and low GIRK1/2 density, where Gα:GIRK ratio slightly exceeded 2 (S6B Fig, low surface density, black bar). Interestingly, for a significant Gβγ-independent intrinsic activity (10% of P_o,max_), up to ~60% of macroscopic I_basal_ could be Gβγ-independent, especially at low channel densities which are common in native cells (S6C Fig). This finding may be relevant to some cells. For instance, coexpression of the Gβγ scavenger phosducin did not significantly reduce I_basal_ in atrial cardiomyocytes [94], where the predominant channel is GIRK1/4.

Finally, we considered the possible impact of variation in the presumed width (W) of the submembrane space within which the GIRK-Gβγ interactions occur. S6D and S6E Fig) shows that the main conclusions regarding the functional stoichiometry of GIRK, Gα and Gβγ remained largely unchanged over a wide range of W, 1–20 nm.

### Activation of GIRK1/2 by coexpression of Gβγ: experiment and simulation

We next tested the ability of the model to predict a new result: the dose dependency of activation by Gβγ, using the estimates of available Gα and Gβγ calculated from basal and agonist-evoked currents. We injected increasing amounts of wt-Gβγ mRNA into oocytes expressing GIRK1/2 at a constant density, and monitored both GIRK currents and Gβγ expression.

**Fig 6:**
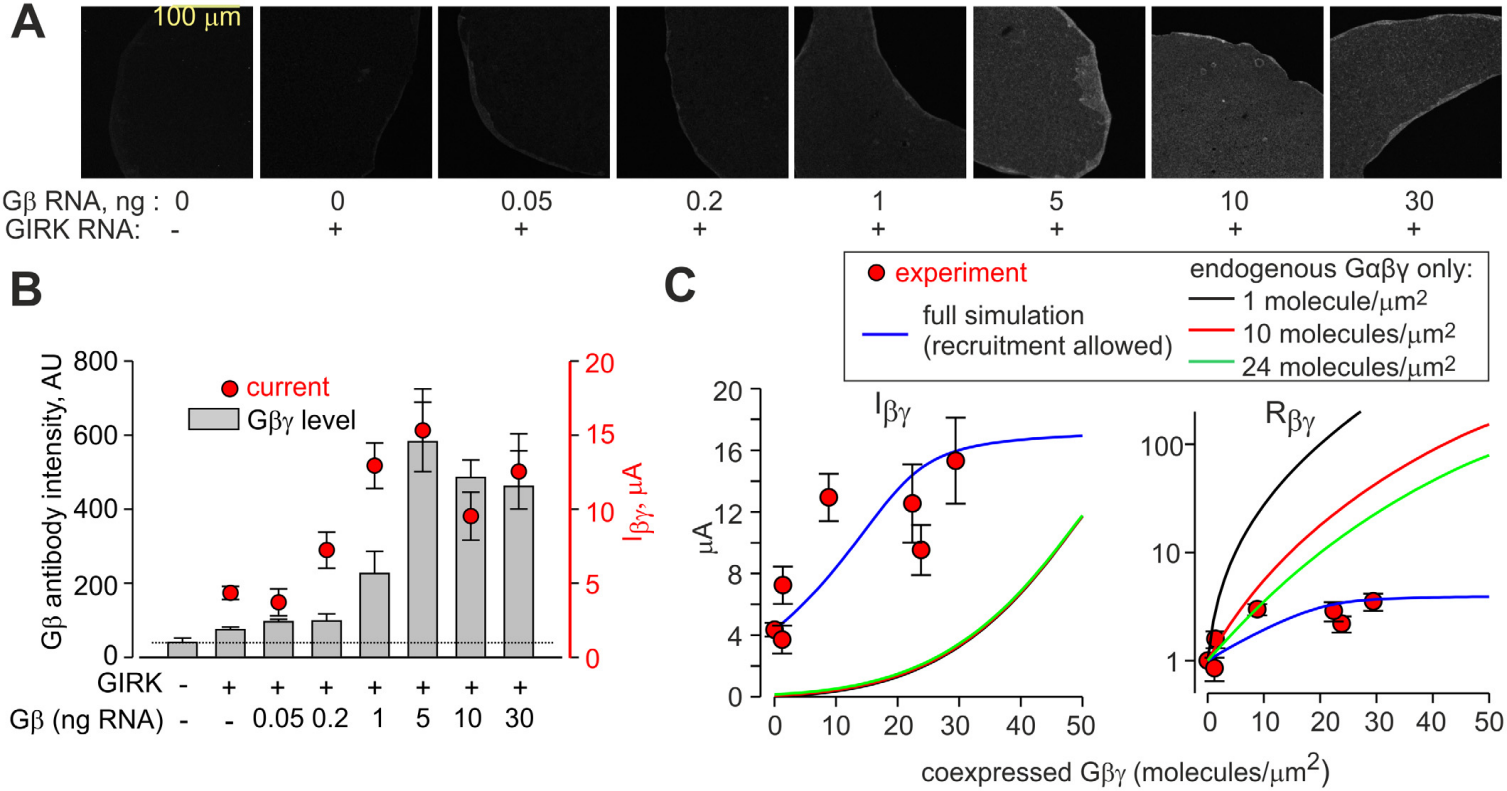
Dose-dependent activation of GIRK1/2 by coexpressed Gβγ: experiment and simulation. GIRK1/2 was expressed at 0.2 ng RNA. All data are mean ± SEM from one experiment. **(A)** Confocal images of Gβγ in giant excised plasma membranes stained with the anti-Gβ antibody. The intensity of all images was increased equally for a better viewing in this figure, but not in the process of image analysis. **(B)** Dose-dependence of Gβγ levels and I_βγ_ in oocytes injected with incrementing amounts of wt Gβγ RNA (0.05–30 ng per oocyte). Gβγ expression in the PM (grey bars) was measured from images shown in A, in 4–8 oocyte membranes, and I_βγ_ currents (red circles; right Y-axis) were measured in 12–16 oocytes. The dashed line shows the basal level of fluorescence, arising from the endogenous Gβγ. Note that, unlike in Western blots, in immunocytochemistry the antibody poorly recognized the endogenous Gβγ compared to the expressed bovine Gβγ. **(C)** Comparison of measured I_βγ_ and R_βγ_ (red circles) and simulated currents (curves). The relative Gβγ levels (from grey bars in B) have been converted into surface densities assuming that 5 ng Gβγ gives 30 molecules Gβγ/μm^2^. The blue line presents the simulation using graded contribution model and amounts of Gα and Gβγ (prior to coexpression of Gβγ) calculated using the methods described above: channel density was calculated from I_βγ_ (13.75 channels/μm^2^ with 5 ng Gβγ RNA in this experiment), and Gβγ and Gα were estimated from I_total_ and I_basal_, giving 3.16 and 0.73 Gβγ:GIRK and Gα:GIRK ratios, respectively. For simulation with endogenous G proteins only and no Gβγ recruitment allowed (red, black and green lines), the channel density was the same and 1, 10 or 24 endogenous Gαβγ were assumed to be available for GIRK1/2.

Relative levels of Gβγ in the PM were directly measured in giant membrane patches of the oocytes [30,95] (Fig 6A) using the anti-Gβ antibody. Absolute surface densities of the expressed Gβγ (X axis in Fig 6C) were calculated assuming that 5 ng mRNA of Gβγ gives 30±4 molecules/μm^2^ (n = 47 oocytes). This density was calculated based on 3 experiments performed during the same period as the experiments of Fig 6 and S8 Fig, with wt-Gβ and YFP-Gγ, and using YFP-GIRK1/2 as the molecular ruler.

As shown in Fig 6B, expression levels of Gβγ in the PM (grey bars) reached maximum at 5 ng RNA/oocyte. I_βγ_ (red circles) reached maximum already at 1 ng Gβγ RNA. Thus, maximal activation of GIRK1/2 has been attained already at submaximal expression levels of Gβγ (see also S8 Fig). Channel density of 13.75 channels/μm^2^ was calculated based on I_βγ_ measured after expression of 5 ng RNA of exogenous Gβγ. Gβγ and Gα available to GIRK without the coexpression of exogenous Gβγ were calculated from I_basal_ and I_total_ (as in Fig 5), yielding ~43 molecules of Gβγ and 10 molecules Gα per μm^2^. We remind that the high density of available “endogenous” Gβγ in the presence of GIRK1/2 is due to Gβγ recruitment, explaining the high I_basal_ and the relatively low index of activation of GIRK by Gβγ in a given oocyte, R_βγ_. (R_βγ_ was defined as I_βγ_/[average I_basal_], where average I_basal_ was determined in a group of oocytes of the same experiment which expressed the channel without Gβγ. The definitions are as in [51]. See S2 Fig for definition of R_βγ_).

Using the calculated GIRK1/2 density and the amounts of available Gα and Gβγ before coexpression of Gβγ, we next calculated the predicted I_βγ_ and R_βγ_ for a range of doses (surface densities) of exogenously coexpressed Gβγ (Fig 6C). The predicted dose-dependencies of I_βγ_ and R_βγ_ (blue lines) are in agreement with experimental data (red circles). Assuming that 5 ng/oocyte of Gβ RNA gives either less (20 Gβγ/μm^2^) or more (44 Gβγ/μm^2^) molecules of coexpressed Gβγ instead of 30 Gβγ/μm^2^ produced similar predictions, still in good agreement with experiment (S7 Fig). Thus, the results of the simulations are relatively insensitive to a 50% variation in our estimate of coexpressed Gβγ. Further, very similar results were obtained in a separate experiment using a different experimental design, where we expressed increasing doses of Gβγ-YFP and calibrated Gβγ-YFP density using YFP-GIRK1/2 as molecular ruler (S8 Fig).

We note that, because channel’s density is estimated from I_βγ_ obtained with a saturating dose of Gβγ, the good agreement between measured and predicted I_βγ_ at this RNA dose might be expected. However, the densities of Gα and Gβγ available to GIRK before coexpression of exogenous Gβγ (0 point on X-axis in Fig 6C) are calculated from I_basal_ and I_evoked_. Therefore, in both experiments (Fig 6 and S8 Fig), the satisfactory predictions of I_βγ_ and R_βγ_ at intermediate Gβγ doses, or the shape of the dose-response curves of I_βγ_ and R_βγ_ vs. Gβγ density, do not result from circular reasoning and are not trivial. This is illustrated by showing simulations that assume equal amounts of endogenous Gα and Gβγ (1, 10 or 24 molecules/μm^2^) available for GIRK1/2, and no Gβγ recruitment. The use of these “classical” assumptions failed to reproduce the experimental data (black, red and green lines in Fig 6C and S7 Fig). In particular, saturation of R_βγ_ was predicted to happen at much higher doses of coexpressed Gβγ than in the experiment, obviously because the presumed initial basal level of Gβγ available to the channels was too low, thus requiring expression of more additional Gβγ.

### Application of the model to the HEK293 expression system and hippocampal neurons

**Fig 7:**
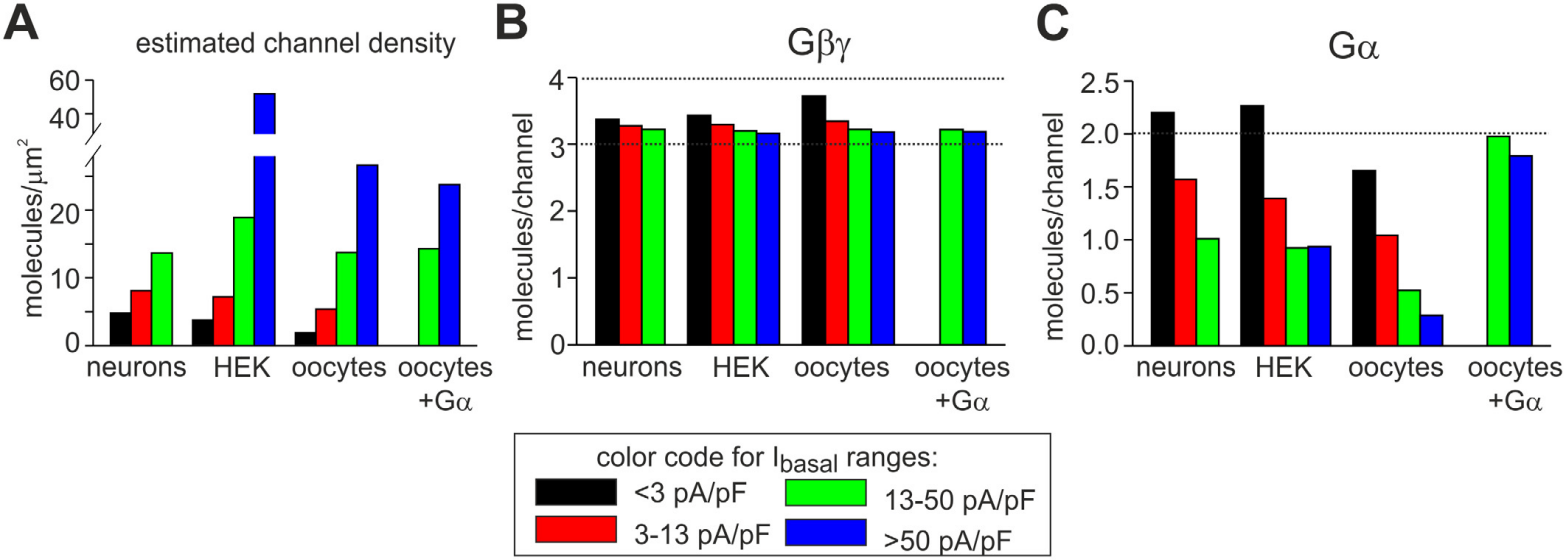
Estimated densities and calculated functional stoichiometries of the GIRK channel, Gβγ and Gα_i/o_ in oocytes, HEK293 cells and neurons. Comparison of cultured mouse hippocampal neurons, and in oocytes and HEK293 cells expressing GIRK1/2. **(A)** Cells were subdivided into four groups according to the indicated I_basal_ ranges, and channel densities were estimated assuming I_βγ_ = 2I_total_ and P_o,max_ = 0.105. Densities in Gα expression experiments in oocytes were estimated from I_total_ in control groups of oocytes expressing GIRK1/2 and m2R only. **(B, C)** Estimates of Gβγ and Gα available for GIRK activation in the 4 channel density groups. In oocytes and HEK293 cells I_evoked_ was elicited by ACh via m2R, in neurons—by baclofen acting on GABA_B_ receptors.

We next evaluated the model’s applicability to another expression system (HEK293 cells), and also to hippocampal neurons that natively express GIRK1/2 channels. We re-grouped raw data previously obtained in HEK cells expressing m2R and GIRK1/2 [51] (Table 2), the data from cultured hippocampal neurons (Fig 1), and the data obtained in oocytes, in a uniform manner. To enable direct comparison between the different systems, we arbitrarily segregated all the recordings into 4 groups, based on basal GIRK currents: <3 pA/pF, 3–13 pA/pF, 13–50 pA/pF and > 50 pA/pF (Table 4). For modeling, we needed to estimate channel surface densities, which have not been directly measured in HEK293 cells and neurons. To this end, we used I_total_ to indirectly assess the channel densities. The ratio I_βγ_/I_total_ is fairly consistent in oocytes and HEK cells, ranging between 1.6 and 2.2 (Table 1 and ref. [96]). Thus, for each I_basal_ range, we calculated I_βγ_ from I_total_ assuming I_βγ_/I_total_ = 2 (Fig 7). Then we calculated densities using Eq 2 and P_o,max_ of 0.105, as measured in oocytes.

**Table 4:** GIRK currents in mouse hippocampal neurons and in GIRK1/2-expressing *Xenopus* oocytes and HEK293 cells. Data are presented as mean ± SEM. Current amplitudes in HEK293 cells and neurons were adjusted to 24 mM K^+^ (as in oocytes) as described in Methods.

I_basal_ range, pA/pF	corresponding I_basal_ in oocytes	cell type	I_basal_, pA/pF	I_evoked_, pA/pF	I_total_, pA/pF	R_a_	n
0.5–3	0.1–0.6 μA	neurons	1.8±0.1	13.5±2.3	15.3±2.3	9.4±1.3	25
		oocytes	1.82±0.1	4.2±0.4	6.0±0.4	3.5±0.3	29
		HEK cells	1.51±0.2	10.5±3	12.03±3	8.3±2	8
3–13	0.6–2.6 μA	neurons	5.8±0.4	19.9±2.3	25.7±2.3	4.8±0.5	29
		oocytes	7.2±0.4	10±0.8	17±0.9	2.5±0.1	74
		HEK cells	6.4±1.1	16±7	22.8±6.7	4.3±1.7	5
13–50	2.6–10 μA	neurons	17.3±2.2	26.1±6.5	43.4±6.2	2.7±0.5	6
		oocytes	28±0.9	15.6±1	43.6±1.3	1.6±0.04	128
		HEK cells	25.7±3.1	34±7	60±9	2.4±0.2	9
>50	>10 μA	neurons	-	-	-	-	-
		oocytes	65.9±2.1	84.5±2.7	84.5±2.7	1.3±0.03	41
		HEK cells	67.6±4.6	96±28	164±26	2.5±0.5	3

The segregation of GIRK activity by I_basal_ yielded relatively similar channel density groups in all cell types; neurons did not appear to express large amounts of GIRK, and the high density group was empty (Fig 7A). Next, using the procedure of Fig 5, we calculated the Gβγ and then Gα available for GIRK in all cases (Fig 7B and 7C). In all three systems, the relationship between G protein subunits and channel density was very similar to that found in oocytes. The most striking result was the persistent, channel density-independent availability of more than 3 Gβγ molecules per channel (Fig 7B). In all cases, the estimated Gα:GIRK ratio was about 2 for the low channel densities, but this number decreased as channel density increased (Fig 7C). Nevertheless, estimates of Gα:GIRK ratio in oocytes were lower than in HEK cells or neurons, indicating that there was a relative shortage of GIRK-associated Gα_i/o_ in oocytes. We have therefore reanalyzed the experiments [30] in which Gα was co-expressed in oocytes in 5–10 fold excess (in terms of RNA quantities) over GIRK1/2 (Fig 7, denoted as “oocytes+Gα”). These doses produced the maximal GIRK-specific "priming" effect of Gα_i3_: strong reduction in I_basal_ and increase in I_evoked_ without a significant reduction in I_total_ (S1 Table). To note, coexpression of Gα_i3_ also produced R_a_ of ~10 which was comparable to the neurons with the lowest I_basal_ and highest R_a_ (compare S1 Table and Table 4). Calculation of available Gβγ and Gα showed a robust persistence of Gβγ:GIRK ratio of above 3 (Fig 7B). Expectedly, the estimate of the available Gα greatly increased after Gα_i3_ overexpression, but, remarkably, Gα:GIRK ratio did not exceed 2 Gα molecules/channel (Fig 7C).

Variations in I_βγ_/I_total_ ratio and P_o,max_ in different cells could bias our estimates of channel density (Eq 2) and thus also estimates of Gβγ and Gα. Therefore, for neurons, we repeated our calculations for a range of I_βγ_/I_total_ ratios between 1.5 and 3 (S9A Fig) and P_o,max_ between 0.05 and 0.2 (S9C Fig). For comparison, a similar range of I_βγ_/I_total_ ratios was also tested for the oocyte data (S9B Fig). The exact values of Gβγ:GIRK and Gα:GIRK ratios varied, especially with changes in I_βγ_/I_total_ ratio. Generally, the lowest channel density is most sensitive to perturbations, and, for I_βγ_/I_total_ = 1.5 (the lowest ratio tested), calculated Gβγ:GIRK and Gα:GIRK ratios exceed our usual estimates. However, this ratio is lower than that observed experimentally (Table 1), likely causing an overestimate of the values of Gβγ and Gα. In all, although the absence of direct measurements of channel densities and P_o,max_ in HEK cells and neurons introduces an element of uncertainty, our results support the functional stoichiometry of 3–4 Gβγ and 2 or less Gα molecules per GIRK1/2 channel. Importantly, for a wide range of parameters, the main trends persist: available Gβγ is in excess over Gα; Gβγ:GIRK ratio remains high (>3) whereas Gα:GIRK ratio decreases as I_basal_ increases.

### Changes in functional stoichiometry of GIRK, Gβγ and Gα explain the inverse R_a_-I_basal_ correlation

**Fig 8:**
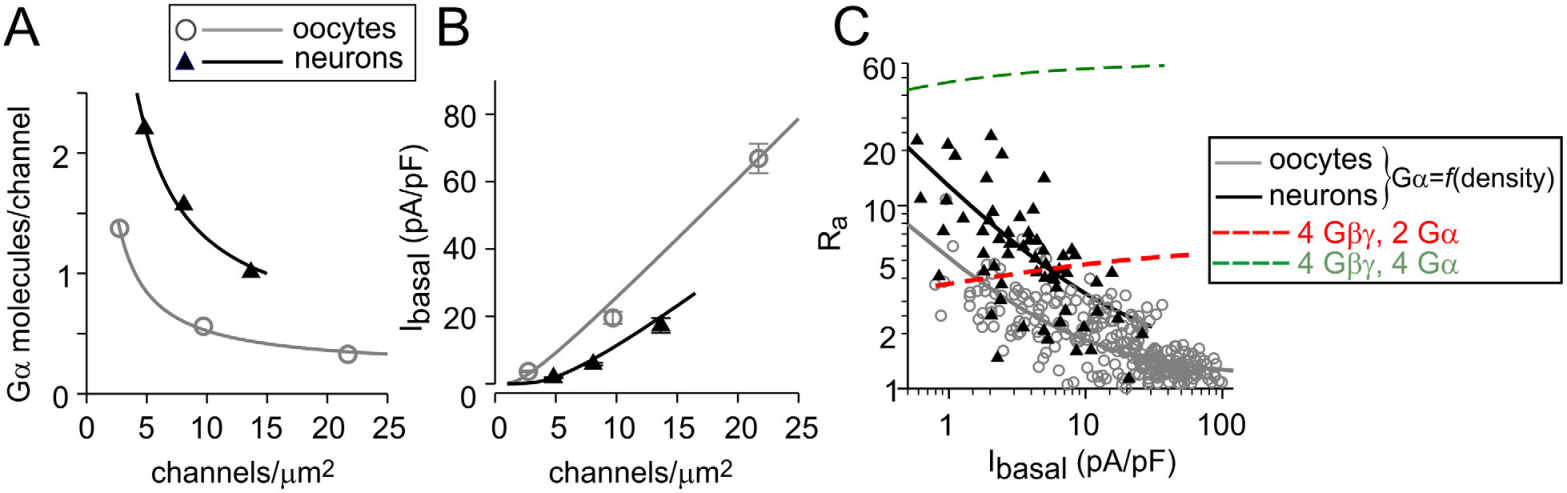
Inverse relation between I_basal_ and R_a_ arises from the decrease in Gα available for GIRK activation at higher I_basal_. **(A)** Gα molecules/channel as a function of channel density. Data for Gα:GIRK and channel density were adopted from Tables 1 and 3 (oocytes) and Table 4 (neurons). To generate a continuous curve, the channel density-Gα relationship was arbitrary fitted with a hyperbolic decay function of the form Gα = Y_o_ + *a*/x, where x is channel density and *a* is a constant. **(B)** Simulated relation between I_basal_ and channel density. We utilized eqs 5–15 and solved them numerically in the 1–30 channels/μm^2^ range, using constant values of Gβγ:GIRK ratio (3.5 for oocytes and 3.4 for neurons) and the calculated values of Gα:GIRK from the fitted curves shown in A. **(C)** Simulated relationship of I_basal_ and R_a_, with variable Gα:GIRK (from A) and constant Gβγ:GIRK ratios. Simulations with 4 Gβγ and 2 Gα (red line) or 4 Gβγ and 4 Gα (green line) available for one GIRK1/2 channel at all densities did not adequately describe the data.

The systematic study presented above supports our hypothesis [51] that the inverse R_a_-I_basal_ relationship for GIRK1/2 reflects a progressive decline in GIRK1/2-associated Gα relative to Gβγ. We could now test this hypothesis quantitatively, and establish whether the calculated changes in GIRK:Gα:Gβγ functional stoichiometry can fully account for the R_a_-I_basal_ relationship shown in Fig 1. To this end, we simulated the changes in R_a_ as a function of I_basal_ for a range of channel densities. We used channel densities, I_basal_ values and Gβγ:GIRK and Gα:GIRK ratios calculated above (Tables 1 and 3 for oocytes, Table 4 for neurons). No free parameters were allowed. The results are shown in Fig 8, for oocytes (grey circles) and hippocampal neurons (black triangles). At this point, the channel density estimates and thus the simulations for oocytes are more reliable than for neurons.

First, for simplicity, we assumed a constant Gβγ:GIRK ratio at all densities (3.5 in oocytes and 3.4 in neurons; see Fig 7). For further simulations, in order to construct continuous curves, we needed to assign numeric values for Gα:GIRK ratios within the full range of channel densities, based on the individual data points calculated for the “density groups” (Fig 8A). Since the reduction in Gα:GIRK ratio as a function of channel density is a process of unknown nature, the data points were arbitrarily fitted to a hyperbolic function (Fig 8A, solid lines). Next, we simulated the relation between I_basal_ and channel density, by substituting the obtained values of Gα:GIRK into eqs 5–15 (Fig 8B). The simulation gave a good match to data of Table 1 (oocytes) and Table 4 (neurons), indicating that the fitting procedure of Fig 8A was satisfactory. Finally, values of Gα:GIRK from Fig 8A and I_basal_ from Fig 8B were used to simulate the R_a_-I_basal_ relationship (Fig 8C, solid black and grey lines), matching well the raw data (triangles and circles). Simulations based on a constant relations of GIRK, Gα and Gβγ at all channel densities (allowing Gβγ and Gα recruitment) could not account for the observed trend in R_a_ changes. This is exemplified for a 1:4:2 and 1:4:4 GIRK:Gβγ:Gα stoichiometry (Fig 8C, red and green dashed lines, respectively). We conclude that the decrease in Gα available for GIRK activation at higher I_basal_ can fully account for the inverse R_a_-I_basal_ relationship in both experimental systems.

## Discussion

### General summary

In this work we have quantitatively analyzed the GPCR-G_i/o_-GIRK1/2 cascade, focusing on basal (I_basal_) and agonist-evoked (I_evoked_) activities, both of which regulate neuronal excitability. We developed a mathematical model which allows quantification and simulation of macroscopic GIRK1/2 currents under steady-state conditions, before and after activation by neurotransmitter or by Gβγ. Our simulations fully rested on experimental data and parameters obtained in this and previous works. The modeling accurately described basal and evoked GIRK1/2 currents in two expression systems and in hippocampal neurons in a wide range of channel’s surface densities, correctly predicted the dose-dependent activation of GIRK1/2 by coexpressed Gβγ in *Xenopus* oocytes, and fully accounted for the inverse correlation between I_basal_ and agonist activation index (R_a_) previously observed in heterologous systems and, as shown here, also in hippocampal neurons. Our experimental findings and the model lay the basis for further analysis of the GPCR-G_i/o_-GIRK cascade, for example for GIRKs of different subunit composition, and in different cells.

Importantly, the present quantitative analysis provides novel and often unanticipated insights into the mechanisms of GIRK regulation by G protein subunits, Gβγ and Gα^GDP^. It reveals an unequal and, moreover, variable functional GIRK1/2:Gβγ:Gα stoichiometry: 1) Under all conditions tested, between 3 and 4 Gβγ dimers are available for GIRK1/2; 2) Only two or less Gα are available per GIRK1/2 channel; 3) Increase of GIRK1/2 surface density is accompanied by a proportional increase in Gβγ (which is recruited by the channel), but not Gα. The unequal, effector-dependent Gα-Gβγ stoichiometry within the GIRK1/2 signaling cascade is an unexpected departure from classical schemes which usually assume that, prior to GPCR activation, the heterotrimeric G proteins available to the effector exist as stoichiometric complexes of Gα and Gβγ [97]. We propose that the unique functional stoichiometry of GIRK1/2 with G protein subunits, and the cooperative nature of GIRK gating by Gβγ, underlie the complex pattern of basal and agonist-evoked activities and allow GIRK1/2 to act as a sensitive bidirectional detector of both Gβγ and Gα^GDP^.

**Fig 9:**
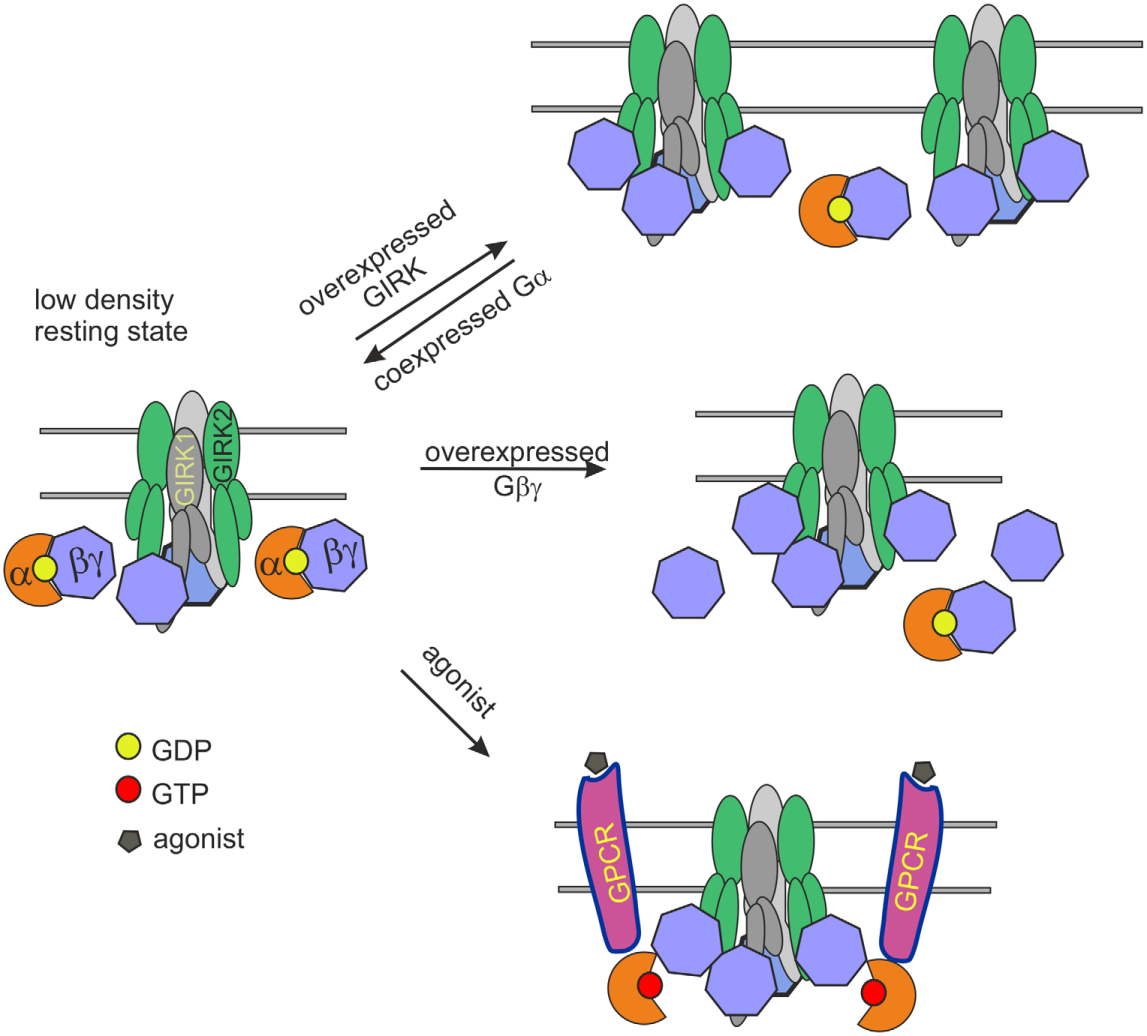
Schematic representation of the GPCR-G-protein-GIRK system. In resting state (no activated GPCR), the GIRK1/2 channel, a heterotetramer of 2 GIRK1 (grey) and 2 GIRK2 (green) subunits, is expected to interact with ~ 3–4 Gβγ subunits, two of which are bound to Gα^GDP^ subunits (GDP is shown by a yellow circle). For simplicity, the hypothetical Gβγ anchoring sites (which may be separate or partly overlapping with the Gβγ-activation sites) are not shown. The interaction of GIRK with Gβγ subunits is reversible. Gα^GDP^ can release the bound Gβγ in basal state, but since Gβγ-Gα^GDP^ interaction is of a high affinity, the probability of GIRK activation due to this process is relatively low. Thus, at any given time the channel is occupied by 2–3 Gβγ molecules (with an open probability of 6–26% of P_o,max_ as shown in Fig 2C). GIRK overexpression leads to a decrease in GIRK:Gα ratio but does not change the GIRK:Gβγ ratio due to the additional recruitment of Gβγ by GIRK1/2, thus effectively increasing the proportion of channels occupied by > 3 Gβγ molecules, leading to an increase in “basal” open probability. The opposite process occurs upon overexpression of Gα, leading to a decrease in free Gβγ available for channel activation. On expression of Gβγ, its availability for channel activation increases, leading to higher fraction of 4 Gβγ-occupied channels with an open probability close to P_o,max_. Activation of G-proteins by an agonist (grey pentagon) via a GPCR (magenta) leads to an exchange of GDP to GTP (red circle) on Gα molecules, and to the subsequent dissociation of the Gαβγ heterotrimer, liberating additional Gβγ for channel activation.

Our conceptual model of GIRK1/2 regulation (Fig 9) rests on the main findings of this study regarding the GIRK1/2:Gβγ:Gα stoichiometry (points 1–3 above) and the notion that, for Gβγ to activate GIRK, it must have its Gα-interacting interface exposed and free to contact GIRK [19,92,98,99]. In the resting state, the channel’s environment is enriched in 3–4 molecules of Gβγ and 1–2 Gα. In this scenario, between one and three Gβγ molecules are not associated with Gα^GDP^ and can bind and activate GIRK, resulting in a basal activity that is between 1 and 26% of total P_o,max_ (see Fig 2C). Because of the gating cooperativity, occupancy of the first two Gβγ-binding sites yields low I_basal_. The fewer Gα, the more “free” Gβγ remains to occupy the activation sites at rest, yielding higher I_basal_. After GPCR-induced separation of Gα^GTP^ from Gβγ (lower arm of the scheme), due to gating cooperativity, addition of each Gα-free Gβγ ensures a robust 4–6 fold activation (e.g. going from 2 to 3 or 3 to 4 Gβγ-occupied sites). An even stronger activation takes place with a shift from 2 to 4 Gβγ-occupied sites (×16); and so on. Overexpression of Gβγ “sequesters” Gα and allows full occupancy of all Gβγ binding sites (middle arm of the scheme). Finally, overexpression of GIRK1/2 recruits Gβγ but not Gα, increasing Gβγ/Gα ratio (upper arm of the scheme). The balance between available Gα^GDP^ and Gβγ yields a continuum of basal activity magnitudes even on the level of a single channel, and sensitively regulates the extent of activation by the agonist.

The scheme emphasizes the important fact that, given the relatively fixed amount of Gβγ available for GIRK1/2 activation, it is the availability of Gα_i/o_ that determines the level of basal activity and, consequently, the extent of activation by agonists (as experimentally observed previously; [30,31]). The imbalance between Gβγ and Gα renders GIRK1/2 with a sizeable I_basal_, allowing it to act as a bidirectional, servo-like device [51] where its activity can be regulated not only by positive (Gβγ, Na^+^, PIP_2_) but also negative (Gα^GDP^, protein kinase C, Gα_q_^GTP^, PIP_2_ depletion) stimuli.

### Surface levels of GIRK and G proteins in *Xenopus* oocytes

Surface levels of endogenous G protein subunits and of heterologously expressed channels and G proteins crucially determine the behavior of the reconstituted signaling cascade, but they have never been quantitatively studied in the past in this common model system. We obtained very close estimates of surface levels of GIRK using two independent approaches: quantitative immunochemistry (which measures all channels in the PM) and electrophysiology (which counts only functional channels) (Fig 4). This indicated that the majority of GIRK1/2 channels in the PM of *Xenopus* oocytes were functional. Further use of GIRK1/2 as a molecular fluorescent ruler for Gβγ yielded Gβγ density very close to density estimated from quantitative immunochemistry, affirming the “molecular ruler” procedure with GIRK1/2 and lending additional support to the measurements of GIRK1/2 density.

We estimated total cellular endogenous Gβγ in the oocyte as ~170 nM, similar to other cell types, 200–800 nM [100] and to the recent high-precision mass spectrometry measurement of ~200 nM Gβ in Xenopus eggs [101]. Notably, examination of data reported in the latter work [101] suggests a total concentration of all Gα in *Xenopus* eggs of ~350 nM. Thus, total oocyte's Gβγ is not in excess over Gα, supporting our assumption (S4 Fig) that endogenous heterotrimeric G proteins are in the Gαβγ form before activation by GPCRs or coexpression of GIRK.

Our estimate of 24 molecules/μm^2^ of endogenous PM-associated Gβγ, presumably as Gαβγ, is comparable to the ~40 molecules/μm^2^ evaluated in HEK cells [102]. In terms of concentration, 24 molecules/μm^2^ corresponds to ~4 μM, much higher than the cytosolic level of ~0.2 μM. Such enrichment of G proteins at the PM is expected, because of the lipid modification of both Gα and Gγ [84,103]. It is probable that a substantial fraction of the PM-associated endogenous Gβγ is not available for GIRK activation, being associated with Gα_s_ or Gα_q_ rather than Gα_i/o_, or located in separate PM compartments, or associated with other effectors such as adenylyl cyclase [104]. Simulations showed that the main conclusions of our study are not affected by assuming a wide range of endogenous G_i/o_ proteins available for GIRK, from 1 to 24 molecules/μm^2^.

In this work, we varied the levels of heterologously expressed GIRK1/2 and Gβγ. For GIRK1/2, the surface densities ranged from about 1 to 30 molecules/μm^2^ in oocytes and about 1–60 channels/μm^2^ in HEK293 cells (Table 4 and Fig 7). Densities of <20 channels/μm^2^ were estimated in cultured hippocampal neurons (Fig 7A). This is comparable to 9–10 channels/μm^2^ found in spines of cerebellar Purkinje neurons by quantitative electron microscopy [105]. Thus, the "low" and "intermediate" densities of expressed GIRK1/2 and Gβγ in oocytes and HEK293 cells may be the most physiologically relevant.

### Modeling GIRK1/2 regulation by Gβγ in basal and agonist-activated states

For our description of GIRK currents, we have considered several models of GIRK channel gating by Gβγ (Fig 2B): the previously developed "concerted activation" model [58], the”graded contribution” model, and a more general model that includes 4 to 5 closed-open transitions. We were able to show that the latter two models converge to the same form of description of macroscopic steady-state P_o_ based on fractional contributions of channels occupied by 1 to 4 Gβγ molecules, experimentally demonstrated for GIRK1/4 [13,14] (see Results and Supplemental Discussion S2 Text for a detailed discussion). We have therefore chosen the graded contribution model as our main tool to simulate and predict GIRK1/2 currents. Despite its relative simplicity, this model incorporates several complex properties of GIRK gating, and provides a strong computational tool for the analysis of the G protein-GIRK signaling. First, in this model we implemented the gating cooperativity of GIRK, by including the graded contribution of each bound Gβγ to channel opening (Fig 2C). Second, to our knowledge, this is the first model to describe both basal and agonist-evoked GIRK activity. Third, the model allows to estimate the amount of G protein subunits available for channel activation without any *a priori* assumptions regarding the levels of endogenous Gα or Gβγ; the GIRK-available Gα and Gβγ are calculated from experimental data (Fig 5). Finally, our method of calculating the functional stoichiometry of GIRK and Gβγ applies even if I_basal_ is partly due to the presence of a GPCR activated by low dose of an ambient neurotransmitter. In this case, the available Gα calculated using the method of Fig 5 will represent only that fraction of GIRK-coupled Gα that is still in its GDP-bound form.

In this work we left aside auxiliary/modulatory proteins such as RGS, focusing on the minimal essential composition of the cascade. We also have not addressed the impact of direct GIRK-Gα interaction. We and others did not find significant direct effects of Gα_i_^GDP^ on GIRK1/2 gating; the main function of Gα^GDP^ is the prevention of basal activation of the channel by ambient Gβγ and the release of Gβγ for channel activation by agonist/GPCR [31,34,36,106]. This function is fully implemented in our model. As for Gα_i_^GTP^, it regulates the kinetics of I_evoked_ of GIRK1/2 but barely affects the steady-state amplitude [29,36]. Our present results suggest that, for GIRK1/2, effects of activated Gα^GTP^ on GIRK1/2 current amplitude are negligible, within a possible ~10% error. These considerations justify the omission of Gα-GIRK binding reactions from model’s equations. It remains possible that certain Gα^GTP^ may differently regulate GIRK channels of other subunit compositions or under certain conditions [34,107].

Both graded contribution and concerted models showed that I_basal_ and I_evoked_ of the expressed GIRK1/2, and their changes with channel surface density, cannot be accurately described unless there is a recruitment of Gβγ to the PM by the channel. Recruitment of Gα is probably negligible (S4 Fig, Table 3). The prediction of preferential availability of Gβγ over Gα for GIRK1/2 concurs with the experimental findings [25], supporting the model’s validity. Further validation came from predicting the system’s response to perturbation in the form of dose-dependent response to coexpression of Gβγ, producing a satisfactory simulation of both I_βγ_ and R_βγ_ (Fig 6, S7 and S8 Figs). Finally, on the basis of experimental data and the calculated molar ratios of GIRK:Gβγ:Gα for different channel densities, the model fully accounted for the inverse correlation between R_a_ and I_basal_ in GIRK1/2 (Fig 8), which was the starting point of this endeavor.

We also made the first steps to extend the model to hippocampal neurons. The analysis of GIRK behavior in HEK cells and hippocampal neurons yielded estimates of Gβγ:GIRK and Gα:GIRK ratios close to those obtained in oocytes (3–4 Gβγ and ≤ 2 Gα), and a satisfactory simulation of the inverse R_a_-I_basal_ relation observed in the neurons (Fig 8). Uncertainties remain, because calculation of channel density in neurons relied on oocyte and partly HEK cell data for I_βγ_/I_total_ ratios and P_o,max_. A variety of GIRK compositions and distinct localization and density in cellular compartments further complicate the picture. Future quantitative studies are warranted for a more accurate description of GIRK activity in various neurons.

### Functional stoichiometry of GIRK and G protein subunits

Our analysis provides two major insights into functional stoichiometry of GIRK1/2 vs. the G protein subunits. First, the molar ratios are both uneven and can change as a function of channels’ density in the PM. The stoichiometry of more than 3 Gβγ per GIRK1/2 is practically invariable, whereas the amount of available Gα_i/o_ is lower than Gβγ and further drops sharply as the level of expression of the channel increases. These estimates of Gβγ and Gα availability remained remarkably stable under a wide range of potentially variable parameters, such as K_D_ of GIRK-Gβγ binding, the extent of Gβγ-independent basal activity, the size of submembrane reaction space, the I_total_/I_βγ_ ratio and P_o,max_ in neurons, etc. Second, unexpectedly, the limiting stoichiometry for Gβγ:Gα:GIRK is 4:2:1.

The uneven Gβγ:Gα stoichiometry and the decrease in Gβγ:Gα ratio were suggested by previous qualitative findings [50,51]. Our new results support this hypothesis and provide new insights into the underlying mechanism. Assumptions of pre-assembly of GIRK1/2 with 1, 2, 3 or 4 Gαβγ heterotrimers failed to recapitulate the observed macroscopic currents and R_a_ (S4 Fig). The decrease in Gα/GIRK ratio as channel’s levels increase is consistent with total PM concentration of Gα_i/o_ being relatively constant at all GIRK1/2 densities (Table 3). This is in agreement with little or no recruitment of Gα to the PM by GIRK1/2 [25,50]. On the other hand, the total amount of GIRK-available Gβγ in the PM increases as more GIRK1/2 channels are expressed, substantially exceeding the “basal” concentration of Gβγ of the naïve oocytes. The conspicuous persistence of Gβ:GIRK stoichiometry is best demonstrated in the oocytes, where it rests on a full quantitative analysis of experimental data. The identical estimates obtained in HEK cells and neurons (Fig 7B), though based on partial data, provide further support. The ability of GIRK1/2 to sustain a steady Gβγ-enriched environment strongly argues for a strong association between Gβγ and GIRK1/2, in line with the proposed high-affinity “anchoring” and recruitment of Gβγ by GIRK1 [25]. The mechanism of Gβγ recruitment is unknown but may be due to co-trafficking from the endoplasmic reticulum [22] or "kinetic scaffolding" and similar mechanisms [63,108,109], as discussed in [25]. At present we cannot rule out that the participation of another Gβγ-binding PM protein, such as a GPCR, or unknown scaffolding proteins, is important for the enrichment of Gβγ.

It is widely accepted that the GIRK signaling cascade occurs within signaling complexes of GIRK channels with subunits of G_i/o_ heterotrimeric proteins and some GPCRs (reviewed in [4,61,110,111,112]). The uneven and variable stoichiometry within the GIRK1/2-Gβγ-Gα_i_ signaling complex revealed by this study is compatible with a high-affinity, dynamic complex, where the channel and the G protein subunits are allowed to dissociate and reassociate in the PM [113]. These considerations justify our use of standard kinetic formalism for modeling. Finite affinity and reversibility of GIRK-Gβγ interaction is also supported by the demonstration of competition between two Gβγ effectors, GIRK and voltage-gated calcium channels, for available Gβγ in sympathetic neurons [114], and by recent GIRK2 reconstitution studies in lipid bilayers [106].

In contrast to Gβγ, our present analysis supports the notion [36,51,106] that, at least in heterologous or artificial systems, Gα_i/o_^GDP^ is not an obligatory partner in the complex. Notably, in neurons and HEK cells the calculated Gα:GIRK ratios are higher than in oocytes (Fig 7 and Table 4); thus the higher R_a_ in these cells. The greater availability of Gα in HEK cells and neurons may reflect the presence of scaffolding or trafficking aids absent in the oocytes. However, the inverse R_a_-I_basal_ correlation and the (calculated) reduction in Gα at high channel densities are maintained in neurons and HEK cells, supporting the expendability of Gα.

The apparent limiting stoichiometry of 4 Gβγ molecules per channel is not surprising, since the model explicitly includes 4 Gβγ-binding sites per channel. However, the calculated availability of two or less Gα_i/o_^GDP^, under most conditions examined, was unexpected. The limit of 2 Gα per channel was only slightly exceeded in simulations of lowest channel densities and when allowing substantial deviations from our standard assumptions (S9 Fig). Most conspicuously, overexpression of Gα_i3_, which reduced I_basal_ by 75–80% and elevated the activation index R_a_ to about 10, increased the calculated Gα:GIRK ratio from less than 0.5 to 2—but no more—Gα molecules per channel (Fig 7, S1 Table). Taken together, our results point to a limiting stoichiometry of 2 Gα molecules available for a GIRK1/2 channel. It is not clear what limits the amount of available Gα, but it is tempting to speculate that this limit reflects the maximal number of Gα molecules that can interact with GIRK1/2. The actual stoichiometry of this interaction is unknown, but the NMR study of Shimada and colleagues [115] indicates that the interacting surface of Gα^GTP^ requires two GIRK1 subunits of a GIRK1 tetramer for full contact. One GIRK1 subunit interacts with the helical domain of Gα and the other one with the GTPase domain [115].

We emphasize that our conclusions are valid for GIRK1/2 but may not be so for other GIRK channels. Thus, homomeric GIRK2 channels, with their low I_basal_ and very high response to Gβγ, do not recruit Gβγ to the PM [25] and probably do not show pre-association with 3–4 Gβγ, or may have more Gα. Excess of Gβγ over Gα has been observed in the phototransduction cascade [116,117] though it has not been linked to any effector. We speculate that effector-dependent changes in the balance of Gα and Gβγ may take place with effectors other than GIRK, playing a role in their regulation.

## Materials and Methods

### Ethics statement

All experiments were approved by Tel Aviv University Committee for Animal Use and Care (permits M-08-081 and M-13-002 for *Xenopus* frogs and M-12-061 for mice).

### Animals and oocyte culture

Female frogs, maintained at 20±2°C on 10 h light/14 h dark cycle, were anaesthetized in a 0.17% solution of procaine methanesulphonate (MS222), and portions of ovary were removed through a small incision on the abdomen. The incision was sutured, and the animal was held in a separate tank until it had fully recovered from the anesthesia and then returned to the tank. The animals did not show any signs of postoperational distress and were allowed to recover for at least 3 months until the next surgery. Following the final collection of oocytes, anaesthetized frogs were killed by decapitation and double pithing. *Xenopus* oocytes were injected with RNA, and incubated in for 3–4 days at 20–22°C in NDE-96 solution (in mM: 96 NaCl, 2 KCl, 1 CaCl_2_, 1 MgCl_2_, 2.5 Na-pyruvate, 50 μg/ml gentamycin, 5 mM HEPES/NaOH, pH 7.5). All experiments with nerve cells derived from newborn mice, that have been analyzed in this paper, have been performed previously [47], and no additional mice have been used.

### Antibodies, cDNA constructs, proteins and RNAs

The anti-GIRK1 polyclonal antibody was from Alomone Labs (Jerusalem), #APC-005. This antibody was raised against the distal C-terminal residues 437–501 of mouse GIRK1. 3 μg/mL were used for Western blots. The anti-Gβ polyclonal antibody (T-20, from Santa Cruz. #sc-378) is directed against the last 50 amino acids (a.a.) of mouse Gβ. 0.4 μg/mL were used for Western blots.

Most DNA constructs were as reported previously: bovine Gβ_1_, bovine Gγ_2_, human muscarinic type 2 receptor (M2R), human Gαi3, rat GIRK1, mouse GIRK2, YFP-GIRK1, GIRK2-HA, Gβ_1_-YFP, Gγ_2_-YFP and Gγ_2_-CFP [36,51,57]. To compare antibody labeling efficiency of bovine vs. *Xenopus* Gβ, we created the YFP-Gβ-XL construct in which the last 50 amino acids of the C-terminus in bovine Gβ were made identical to those of *Xenopus* Gβ_1_, by mutating 7 a.a.: V296I, A299C, A302R, D303E, A305V, A309S and D322S by standard PCR protocols. Preparation and storage of Gβ_1_γ_2_ and of the GST-fused distal C-terminus of GIRK1, GST-dCT (a.a.365-501) used for calibrations of Fig 4A were done as described previously [69,118]. RNA was synthesized *in vitro* [57]. Amounts of injected RNA are indicated in the text, Tables 1, S1 and in Figure legends.

### Confocal imaging and calculation of surface density of Gβγ-YFP

Fluorescence levels of the expressed YFP (yellow fluorescent protein) and cerulean (termed here CFP, cyan fluorescent protein) were measured in intact *Xenopus* oocytes essentially as described [25,32]. Both YFP and CFP carried mutations that increase stability and reduce dimerization [32]. Briefly, oocytes were imaged in ND96 solution in a 0.7 mm glass-bottom dish using Zeiss 510META confocal microscope with a 20× air objective. Images were acquired in the spectral mode. CFP was excited at 405 nm and emission was measured at 481–492 nm. YFP was excited at 514 nm and emission was measured at 535–546 nm. Fluorescent signals were averaged from 3 regions of interest (ROI) using Zeiss LSM Image Browser, and averaged background measured at an area outside the cell was subtracted. The average signal from uninjected oocytes was subtracted for final analysis. Saturation of emission measurement was strictly avoided to ensure that the readout of the confocal microscope was linear within the range of measurement. All measurements were made in the linear range of the recording apparatus.

In calculating Gβγ surface density through comparing fluorescent signals from YFP-GIRK and Gβγ-YFP, the fluorescent intensities of the two proteins were compared directly from oocytes of the same batch on the same day, as described in Fig 4D. No correction for non-fluorescent (improperly folded) YFP [119] was needed (assuming that the percent of misfolding was similar in all YFP fusion proteins used here), because the ionic current I_βγ_ (a fluorescence-independent parameter) was used as the basis for GIRK density estimates.

Giant membrane patches of oocyte membrane were prepared and imaged as described [25,95]. For imaging, fixated membranes were immunostained with the anti-Gβ antibody at 1:200 dilution. Cy3-conjugated anti-rabbit secondary antibody (Jackson ImmunoResearch) was used for imaging with the 543 nm laser, and excitation was measured at 566–577 nm. Background fluorescence from an area outside the giant patch was subtracted. For final analysis shown in Fig 6, the signal from uninjected oocytes was subtracted from all groups.

### Biochemistry

Manual separation of plasma membranes from the rest of the oocyte (“cytosol”) was performed as described [81], with modifications. Plasma membranes together with the vitelline membranes (extracellular collagen-like matrix) were removed manually with fine forceps after a 5–15 min incubation in a low osmolarity solution (5 mM NaCl, 5 mM HEPES, and protease inhibitors (Roche Complete Protease Inhibitors Cocktail, 1 tablet/50 ml), pH = 7.5). The remainder of cell (cytosols) was processed separately. First, the nuclei were separated by centrifuge for 10 min at 700×g at 4°C. Plasma membranes and cytosols were solubilized in 35 μl running buffer (2% SDS, 10% glycerol, 5% β-mercaptoethanol, 0.05% Bromophenol Blue, 62.5 mM Tris-HCl pH 6.8) and heated to 65°C for 5 min. Samples were electrophoresed on 12% polyacrylamide-SDS gel, and transferred to nitrocellulose membranes for Western blotting with the various antisera. The signals were visualized using the SuperSignal kit (Thermo) and quantitated using ImageJ software (National Institutes of Health, USA).

### Electrophysiology

#### Macroscopic current recording in neurons

Raw data from primary cultures of mouse hippocampal neurons used in the analysis of Figs 1 and 7 were from whole-cell patch clamp experiments. A subset of data shown (40 neurons out of 60) had been reported previously [47] but the I_basal_-I_evoked_ relation has not been analyzed. The batch solution contained the low-K^+^ bath solution (in mM: 145 NaCl, 4 KCl, 1.8 CaCl_2_, 1 MgCl_2_, 5.5 D-glucose, 5 HEPES/NaOH; pH 7.4), which was replaced to the high-K^+^ solution for GIRK current measurement (in mM: 120 NaCl, 25 KCl, 1.8 CaCl_2_, 1 MgCl_2_, 5.5 D-glucose, 5 HEPES/NaOH; pH 7.4). Both external solutions contained 0.5 μM TTX and 0.5 mM kynurenic acid. Patch pipettes (3–5 MΩ) were filled with intracellular solution (in mM: 130 K-Gluconate, 0 or 6 NaCl, 1 EGTA, 1 MgCl_2_, 10 HEPES, 2 MgATP, 0.3 Tris-GTP, 0.01 Tris-GDP, pH 7.3). Baclofen (Sigma) was added at 100 μM, sTertiapin-Q or rTertiapin-Q (TPNQ; Alomone labs, Jerusalem) at 100–120 nM [120]. We refrained from using Ba^2+^ as GIRK blocker in neurons because of its low specificity [1]. Indeed, 1 mM Ba^2+^ blocked a much greater fraction of the total inward current in high-K^+^ solution (S1 Fig), confirming that additional Ba^2+^ -sensitive channels contribute to total basal conductance in these cells (e.g. [53]). In contrast, we used Ba^2+^ to block the expressed GIRK channels in *Xenopus* oocytes and HEK293 cells, which have negligible intrinsic (endogenous) Ba^2+^-sensitive basal currents (S2 Fig) [120,121].

Current measurements in neurons were done at -70 mV. To correct for the difference in K^+^ driving force when comparing whole-cell currents from neurons and oocytes (for Fig 7 and Table 4), correction to a holding potential of -80 mV was done assuming E_K_ = -37 mV in the 25 mM K^+^ solution. 4 cells (out of 65) with I_basal_<0.5 pA/pF were discarded because the recording was deemed unreliable owing to the low signal-to-noise ratio. One cell was found to be outlier by Grubb's test using GraphPad outlier calculator http://graphpad.com/quickcalcs/Grubbs1.cfm. No correction has been made for the 1 mM difference in [K]_out_ of oocyte's vs. neuronal high-K^+^ solution (24 vs. 25 mM).

#### Macroscopic current recording in Xenopus oocytes

All experiments were done at 20–22°C essentially as described [51]. Data acquisition and analysis were done using pCLAMP (Molecular Devices, Sunnyvale, CA). Whole-cell currents were measured using two electrode voltage clamp in the ND96 (low K^+^) solution and in a high K^+^ solutions (24 mM K^+^, isotonically replacing NaCl in ND96) as shown in S2 Fig. Currents were recorded at −80 mV, filtered at 500 Hz, and sampled at 5 or 10 kHz. Currents in oocytes were converted to densities, in pA/pF, assuming an oocyte's capacitance of 200 nF [88]. For analysis of correlation between I_evoked_ and R_a_ and for Table 1, new raw data in the low and high GIRK1/2 density groups (total of 41 cells) were combined with raw data collected for our previous publication [30] (20 cells). I_basal_ was measured after blocking all GIRK currents by 5 mM Ba^2+^ [31].

#### Patch clamp recordings in Xenopus oocytes

Patch clamp experiments were done using Axopatch 200B (Molecular Devices, Sunnyvale, CA). Currents were recorded at -80 mV, routinely filtered at 2 kHz and sampled at 20 kHz. In some patches we also used filtering at 5 kHz. Patch pipettes had resistances of 1.4–3.5 MΩ. Pipette solution contained, in mM: 144 KCl, 2 NaCl, 1 MgCl_2_, 1 CaCl_2_, 1 GdCl_3_, 10 HEPES/KOH, pH 7.5. GdCl_3_ completely inhibited the stretch-activated channels. The bath solution contained, in mM: 144 KCl, 2 MgCl_2_, 6 NaCl, 1 EGTA, 10 HEPES/KOH, pH 7.5. To obtain single channel recordings, oocytes were injected with low doses of RNA of GIRK1 (10–50 pg), and RNA of GIRK2 was 1/2 to 1/3 of that (5–17 pg), to avoid the formation of GIRK2 homotetramers. In addition, 50 ng of the antisense oligonucleotide against oocyte's endogenous GIRK5 was injected to prevent the formation of GIRK1/5 channels [122]. Number of channels was estimated from overlaps of openings during the whole time of recording (at least 5 min). Single channel current (I_single_) was calculated from all-point histograms of the original records [123], and open probability (Po) was obtained from event lists generated using idealization procedure based on 50% crossing criterion [124]. Po was calculated only from records that contained 1, 2 or 3 channels. Each recording lasted for at least 4 min and contained >10,000 openings. Thus, the probability of missing a channel was negligible (p<10^−248^ for 1-channel records). In support, P_o_ in patches with 2 or 3 channels was similar (0.071±0.02, n = 2, and 0.078±0.021, n = 3, respectively) and even lower than in 1-channel patches (0.15±0.026, n = 3), opposite to what would be expected in the case of underestimation of channel number. GPCR-evoked GIRK1/2 activity was induced via the coexpressed m2R with 2 or 5 μM ACh in the patch pipette. 2 μM ACh is a saturating concentration for GIRK1/2 expressed in *Xenopus* oocytes [62]. Because a slow reduction of activity over several minutes was observed in some patches, ACh-induced P_o_ was estimated during the first minute of the record. For channels activated by coexpressed Gβγ, there was no decrease in P_o_ over >4 minutes, and the P_o_ was averaged from the first 4 minutes of the record.

#### Macroscopic current recording in HEK293 cells

Most of the data on GIRK1/2 expressed in HEK293 cells transfected with cDNAs of GIRK1 and GIRK2 and m2R (Tables 2 and 4; Fig 7) are from experiments described previously [51]. Data on I_βγ_ in HEK cells have been obtained in the same series of experiments but have not been reported previously. HEK293 cells were transfected with cDNAs of GIRK1, GIRK2, m2R (0.5 μg each), without or with the edition of DNAs of Gβ1 and Gα_i3_ (0.2 μg each). Whole cell recordings were performed at -80 mV with patch pipette solution containing, in mM: 130 KCl, 1 MgCl_2_, 5 EGTA, 3 MgATP, 10 HEPES. Low-K bath solution contained, in mM: 140 NaCl, 4 KCl, 1.8 CaCl_2_, 1.2 MgCl_2_, 11 glucose, 2 CdCl_2_, 5.5 HEPES. High-K bath solution contained 90 mM KCl and 54 mM NaCl, the rest was as in low-K solution. To compare with data from oocytes (for Fig 7 and Table 4), the correction factor to adjust for current amplitude difference in 90 mM K^+^ (HEK293) vs. 24–25 mM K^+^ solution (oocytes, neurons) was determined experimentally to be 3.27±0.14 (n = 7; measured in oocytes).

### Modeling of Gβγ activation of GIRK1/2

In general, the macroscopic GIRK1/2 current, I, can be calculated utilizing a modified (Eq 1):

$$\text{I}={\text{ I}}_{\text{single}}\cdot {\text{P}}_{\text{o}}\cdot \text{N}/{\text{f}}_{\text{sc}}$$ (5)

where I_single_ is a unitary current and N is the number of channels [1]. f_cs_ is a solution conversion factor between solutions used for whole-cell (24 mM K^+^) and in cell-attached patches (144 mM K^+^). f_sc_ was estimated as 4.63±0.26 (n = 6) by measuring GIRK currents in the same oocytes in the two solutions, in whole-cell configuration.

For the graded contribution model, we define P_o_ as:

$${\text{P}}_{\text{o}}{\text{=P}}_{\text{o,max}}\cdot \sum_{\text{1}}^{\text{4}}{\text{f}}_{\text{p}}{}_{\text{,x}}\cdot \phi_{x}$$ (6)

where P_o,max_ is the maximal open probability, f_p,x_ is the fraction of Po contributed by x Gβγ-occupied channel (x is an integer between 1 and 4), and the *ϕ*_*x*_ is the fraction of channels in the x Gβγ-occupied state and can be calculated according to:

$$\phi_{\text{x}}=\frac{{\text{[C}}_{\text{x}}\text{]}}{{\text{C}}_{\text{total}}}$$ (7)

where [C_x_] is the concentration of channels in x Gβγ-occupied state and C_total_ is the total channel concentration in membrane. For the concerted model, Eq 6 is reduced to

$${\text{P}}_{\text{o}}{\text{=P}}_{\text{o,max}}\cdot \phi_{4}$$

where *ϕ*_4_ is the fraction of channels with four bound molecules of Gβγ [58].

For the graded contribution model, we calculated values of f_p,x_ based on data described by Ivanova-Nikolova et al. (1998)[13] rendering ~ 0.01, 0.06, 0.26 and 1 values corresponding to 1–4 Gβγ occupied states. Based on mass-action law and Fig 2B, GIRK1/2 channel activity can be described by the following system of eqs (8–12):

$$\left[C_{0}]\cdot [G\beta \gamma ]=\frac{1}{4}K_{D}\cdot [C_{1}\right]$$ (8)

$${\text{[C}}_{\text{1}}\text{]}\cdot [\text{G}\beta \gamma ]=\frac{\text{2}}{\text{3}}{\text{K}}_{\text{D}}\cdot {\text{[C}}_{\text{2}}\text{]}$$ (9)

$${\text{[C}}_{\text{2}}\text{]}\cdot [\text{G}\beta \gamma ]=\frac{\text{3}}{\text{2}}{\text{K}}_{\text{D}}\cdot {\text{[C}}_{\text{3}}\text{]}$$ (10)

$${\text{[C}}_{\text{3}}\text{]}\cdot [\text{G}\beta \gamma ]=4\cdot {\text{K}}_{\text{D}}\cdot {\text{[C}}_{\text{4}}\text{]}$$ (11)

$${[\text{C}}_{\text{0}}{]+[\text{C}}_{\text{1}}{]+[\text{C}}_{\text{2}}{]+[\text{C}}_{\text{3}}{]+[\text{C}}_{\text{4}}{]=\text{C}}_{\text{total}}$$ (12)

where the K_D_ is a dissociation constant of Gβγ and GIRK1/2. For our simulations we routinely used K_D_ = 50 nM as measured in direct biochemical experiments [90], but a range of other values has also been tested as explained in the Results.

For both concerted and graded contribution models, G protein dissociation reaction required for modeling of GIRK1/2 basal activity according to the schemes described in Fig 2B is formulated as:

$${\text{k}}_{\text{on}}\cdot [\text{G}\beta \gamma ]\cdot {[\text{G}\alpha }_{\text{GDP}}{]=\text{k}}_{\text{off}}\cdot {[\text{G}\alpha }_{\text{GDP}}\text{G}\beta \gamma ]$$ (13)

$${\text{G}\beta \gamma }_{\text{total}}{=[\text{G}\beta \gamma ]+[\text{G}\alpha }_{\text{GDP}}{\beta \gamma ]+[\text{C}}_{\text{1}}]+2\cdot {\text{[C}}_{\text{2}}]+3\cdot {\text{[C}}_{\text{3}}]+4\cdot {\text{[C}}_{\text{4}}\text{]}$$ (14)

$${\text{G}\alpha }_{\text{total}}{=[\text{G}\alpha }_{\text{GDP}}{]+[\text{G}\alpha }_{\text{GDP}}\text{G}\beta \gamma ]$$ (15)

where Gβγ_total_ and Gα_total_ are total concentrations of corresponding subunits available for interaction with the channel and k_on_ and k_off_ are association and dissociation constants of G_i_ protein subunits (0.7•10^6^ M^-1^ s^-1^ and 0.0013 s^-1^, respectively [56]). Eqs 8–12, combined with Eqs (13–15) is the most general form of description of a system containing GIRK channel and G proteins. For simulation of agonist-evoked activity, with saturating doses of both GPCR and agonist, we assumed a complete dissociation of G- protein heterotrimer [63,64] and thus Eq 14 is changed to:

$${\text{G}\beta \gamma }_{\text{total}}=\;\left[\text{G}\beta \gamma \right]+\left[{\text{C}}_{1}\right]+2\left[{\text{C}}_{2}\right]+3\left[{\text{C}}_{3}\right]+4\left[{\text{C}}_{4}\right]$$ (16)

Simulations were performed using Matlab and Berkeley Madonna software. Steady-state simulations used in model development and application, as well as in most Figures, were done utilizing Matlab 6.5 function “solve” which is a part of Symbolic Math Toolbox. This function first looks for analytical solution, and if the former is absent, switches to numerical iterative algorithm (“trust region algorithm”, “quasi-Newton algorithm”). MATLAB routines for the calculation of Gβγ and Gα available for GIRK with the graded contribution model are shown in Supplemental Methods (S1 Text).

In several cases we tested a range of arguments to produce continuous curves range of changes in GIRK1/2 currents or their ratios (R_a_, R_βγ_) (Fig 6C, S4, S7 and S8 Figs). Here, in order to reduce calculation time, we utilized Berkley Madonna software which implements 4^th^ order Runge-Kutta method for numerical solution of differential equations (see Supplemental Methods, S1 Text). To assure lack of inconsistencies in calculation, we have compared the Matlab and Berkeley Madonna calculation results for a large number of cases and always obtained the same numbers.

### Statistics

Imaging data on protein expression have been normalized as described previously [125]. Fluorescence intensity in each oocyte or giant membrane was calculated relative to the average signal in the oocytes of the control group of the same experiment. This procedure yields average normalized intensity as well statistical variability (e.g. SEM) in all treatment groups as well as in the control group. Statistical analysis was performed with SigmaPlot 11 (Systat Software Inc., San Jose, CA, USA). If the data passed the Shapiro-Wilk normality test and the equal variance test, two-group comparisons were performed using t-test. If not, we performed the Mann-Whitney Rank Sum Test. Multiple group comparison was done with one-way ANOVA if the data were normally distributed. ANOVA on ranks was performed whenever the data did not distribute normally. Tukey’s post-hoc test was performed for normally distributed data and Dunn’s post-hoc test otherwise. Unless specified otherwise, the data in the graphs is presented as mean ± SEM. Correlation between two parameters (such as basal current and R_a_) was tested using the Spearman correlation test by running this test on raw data using the statistical module of SigmaPlot 11.

## Supporting Information

S1 Text Supplemental Methods. MATLAB routines for the calculation of Gβγ and Gα available for GIRK with the graded contribution model; Calculations of model predictions for a range of parameters using the Berkeley Madonna software.
(DOCX)

S2 Text Supplemental Discussion. Conversion from channel densities to concentrations; GIRK1/2 stoichiometry; Estimating steady-state open probability with the “separate gating transitions” model.
(DOCX)

S1 Table Effect of coexpression of Gα_i3_ on GIRK1/2 currents in oocytes. Data are from 2 to 4 experiments, for each group, shown as mean ± SEM. We did not include experiments with extremely large Gα_i3_ RNA quantities, as expression of higher doses of Gα_i3_ usually reduced I_total_, indicating a general Gβγ scavenging effect rather than priming.
(DOCX)

S1 Fig Block of inward currents in cultured hippocampal neurons by TPNQ and Ba^2+^. **(A)** Ba^2+^ (1 mM) blocks a greater fraction of the total inward current in high-K^+^ solution, compared to TPNQ (120 nM). The experimental protocol was the same as in Fig 1, with the additional step of Ba^2+^ addition after TPNQ. ΔTPN and ΔBa denote the magnitudes (shown by double-speared arrows) of TPNQ- and Ba-blocked currents, respectively. Note that Ba^2+^ blocked a much greater fraction of the total inward current in high-K^+^ solution, most probably of the block of additional Ba^2+^ -sensitive channels present in these neurons. **(B)** Comparison of average TPNQ- and Ba^2+^-blocked currents in 14 cells of one batch of neurons. Statistical significance (p<0.001) was determined using Wilcoxon Signed Rank test (the data did not pass normality test).
(TIF)

S2 Fig GIRK1/2 currents in oocytes. Holding potential was -80 mV, low-K^+^ and high-K^+^ solutions contained 2 and 24 mM K^+^, respectively (K^+^ was replaced for Na^+^). Net GIRK currents were determined by subtracting the current remaining after the addition of 5 mM BaCl_2_. (**A**) I_basal_ and I_evoked_ in an oocyte expressing m2R, GIRK1 and GIRK2. Calculation of R_a_ was done in every cell from its own I_basal_ and I_evoked_. (**B**) I_βγ_ in an oocyte expressing m2R, GIRK1, GIRK2 and Gβγ. Note that adding ACh did not evoke a significant additional GIRK current, suggesting full activation by Gβγ. R_βγ_ was calculated in each cell by dividing its own I_βγ_ by the average I_βγ_ from the control group of the same experiment in which no Gβγ was coexpressed. (C) Expression of m2R in a wide range of doses does not affect I_basal_. 5–8 oocytes have been tested in each group. There were no significant differences between treatments as tested by one-way ANOVA.
(TIF)

S3 Fig Characterization of YFP-labeled GIRK1 and Gβ. **(A, B)** Single channel parameters of GIRK1/2 and YFP-GIRK1/2 channels are very similar. **(A)** Cell-attached records of channel activity expressing the channel and Gβγ (5 ng RNA). **(B)** Comparison of average i_single_ and P_o_. Data are from oocytes of the same batch, recorded during a two-day experiment. **(C, D)** The anti-Gβ antibody similarly recognizes YFP-labeled bovine and *Xenopus* Gβ subunits in Western blots of manually pealed plasma membranes. Data are from 4 separate experiments. For Western blots, 15 to 20 plasma membranes were pooled. For confocal imaging, groups of 3–16 oocytes were examined, and the average fluorescence level was compared with that of YFP-GIRK1/2 (therefore the statistical significance was calculated using paired t-test). The density of the latter was calculated from the measurement of currents as explained in the text. In each experiment, both confocal imaging, current measurement and Western blots of manually peeled membranes were done in oocytes of the same donor. There was a good agreement for surface density estimates of YFP-Gβ-XL from confocal "molecular ruler" measurements and from quantitative Western blots, either in absolute terms as molecules/μm^2^ (**C**) or in relative terms, normalized to estimates of YFP-Gβ in each experiment (**D**). YFP fluorescence can be safely assumed to be independent of the species of fused Gβ (mammalian or *Xenopus*). Therefore, similar estimates of surface density observed from confocal imaging and Western blots suggest that the Gβ antibody used here recognizes the oocyte's endogenous Gβ in Western blots similarly to the coexpressed mammalian (bovine) Gβ_1_.
(TIF)

S4 Fig Simulation of density-dependent changes in whole-cell GIRK1/2 activity. Experimental data (from Table 1) are shown as red circles (mean ± SEM). The simulations of currents and Ra were done using the graded contribution model. **(A)** Testing the hypothesis that the endogenous Gαβγ heterotrimers are the only source of Gβγ for GIRK activation; I_basal_ is due to spontaneous dissociation of Gαβγ into Gα^GDP^ and Gβγ (see Fig 2A). Simulations were performed assuming that only part (1 or 10 molecules/μm^2^, black and red curves) or all (24 molecules/μm^2^, blue curves) endogenous G proteins can donate Gβγ to activate GIRK1/2. Note that no satisfactory description of data can be obtained under any of these conditions. The simulated I_basal_ is too low; for high channel densities, also the full I_evoked_ could not be obtained even assuming that all endogenous Gαβγ (i.e. all 24 molecules/μm^2^) could release Gβγ and activate GIRK. **(B)** Testing the hypothesis that the expressed GIRK1/2 recruits additional endogenous G protein subunits to the PM, e.g. from other cellular compartments. Simulations were done assuming that each GIRK1/2 channel recruits from 1 to 4 G_i/o_ heterotrimers. The recruited Gα and Gβγ were added to the pre-existing endogenous plasma membrane-attached Gαβγ before Gβγ expression. **(C)** Testing the hypothesis that the expressed GIRK1/2 recruits additional endogenous Gβγ, but not Gα, to the PM; the rest was done as in B. Calculations in (B) and (C) assumed 24 molecules/μm^2^ of endogenous G_i/o_ available for GIRK. Similar results were obtained assuming 10 molecules/μm^2^ (data not shown). Simulations as in A-C were also done with the concerted model, yielding similar results (data not shown).
(TIF)

S5 Fig The concerted activation model supports the unequal stoichiometry estimates of Gβγ and Gα available for GIRK1/2. The plots present the calculated amounts of Gβγ and Gα available for GIRK1/2 using the concerted model for a range of K_D_ for the GIRK-Gβγ interaction (5–100 nM), for the three channel density groups of Table 1.
(TIF)

S6 Fig The presence of Gβγ-independent intrinsic activity and the dimensions of submembrane reaction space do not significantly alter the estimates of GIRK1/2-available G proteins subunits. Calculations were done assuming K_D_ = 50 nM for the GIRK-Gβγ interaction. **(A-C),** the impact of Gβγ-independent basal activity. Calculation were done for Gβγ-independent intrinsic activity of a single channel ranging from 1% to 10% of P_o,max_. Available Gβγ (**A**), Gα (**B**) and the Gβγ-independent fraction of I_basal_ (**C**) were calculated for the three channel density groups of Table 1. **(D, E)** Varying the submembrane space thickness in a wide range, 1–20 nm, does not significantly change the estimates of functional stoichiometry of GIRK1/2-Gβγ-Gα.
(TIF)

S7 Fig Simulations of the Gβγ dose-response experiment for a range of assumed Gβγ densities. Because in the experiment of Fig 7 the actual density of Gβγ in the PM has not been directly measured, the calculations of Fig 7C assumed that it was equal to the average density of 30 Gβγ molecules/μm^2^ (with 5 ng RNA), as measured in other 4 experiments done during the same time period. Here, we run simulations as in Fig 7C for 20 or 44 molecules Gβγ/μm^2^ (**A, C**) and compare the result with that of Fig 7C (shown here again in **B** for a direct comparison). The color codes are as in Fig 7: the blue line presents the simulation using graded contribution model and amounts of Gα and Gβγ (without coexpressed Gβγ) calculated as explained in Fig 7 legend, and red, black and green lines show simulation with endogenous G proteins only and no Gβγ recruitment allowed.
(TIF)

S8 Fig Another experiment on dose-dependent activation of GIRK1/2 by coexpressed Gβγ. The presentation in similar to that of Fig 7. Gβ was coexpressed with Gγ-YFP in incremental doses, and with a constant amount (1 ng RNA) of wt GIRK1/2. RNA of Gγ-YFP was always half of that of Gβ RNA, by weight. **(A)** Gβγ-YFP fluorescence levels (grey bars, left Y-axis) and GIRK currents (red circles, right Y-axis) are shown on the same plot. GIRK1/2 density, calculated from I_βγ_ of the 17 ng Gβγ-YFP group, was 13 molecules/μm^2^. In addition, we injected YFP-GIRK1/GIRK2 (5 ng GIRK1-YFP) and measured I_basal_ which was 8.4± 1.1 μA (n = 11), comparable to I_basal_ of unlabeled GIRK1/2 (9.4±0.8 μA). Thus, we assumed the same density of ~13 channels/μm^2^ for labeled and unlabeled channels. Since the YFP-GIRK1/2 gave a fluorescent signal of 1237 ±221 AU (n = 7), this signal was assumed to correspond to 26 YFP molecules/μm^2^. This number was used as the basis of calculations of Gβγ-YFP density for plots shown in B. **(B)** Comparison of measured I_βγ_ or R_βγ_ (red circles) and simulated currents or R_βγ_ (blue curves). The left and right Y-axes are related to I_βγ_ and R_βγ_, respectively. Available Gα and Gβγ (before Gβγ coexpression) were estimated from I_total_ and I_basal_, giving 3.82 and 0.42 molecules/μm^2^ of Gβγ and Gα, respectively.
(TIF)

S9 Fig Estimated stoichiometries of Gα and Gβγ available for GIRK in neurons and oocytes in a range of I_βγ_/I_total_ ratios and P_o,max_. Whereas for the oocytes the actual I_βγ_/I_total_ ratio and P_o,max_ are known, in neurons these parameters are not known. Both parameters affect the calculated channel density and could affect the estimates of stoichiometry. The calculations shown in this Figure demonstrate the same general trend in stoichiometries of GIRK1/2, Gβγ and Gα as we have found in the previous analysis in the oocytes, in a range of I_βγ_/I_total_ ratios (for neurons and oocytes; **A** and **B**) and P_o,max_ (for neurons; **C**). The estimates of Gβγ are around 3-4/channel and relatively independent of I_basal_, and those of Gα are below 2 and drop sharply with the increase in I_basal_. Generally, the lowest channel density is most sensitive to perturbations, and, for the lowest simulated I_βγ_/I_total_ ratio, calculated Gβγ/channel and Gα/channel exceed our usual estimates.
(TIF)
