# Supplementary material for: A Quantitative Model of the GIRK1/2 Channel Reveals That Its Basal and Evoked Activities Are Controlled by Unequal Stoichiometry of Gα and Gβγ
Source: PLoS Comput Biol. 2015 Nov 6;11(11):e1004598. doi: 10.1371/journal.pcbi.1004598 (PMC4636287; doi:10.1371/journal.pcbi.1004598)
Supplement: S1 Text — MATLAB routines for the calculation of Gβγ and Gα available for GIRK with the graded contribution model; Calculations of model predictions for a range of parameters using the Berkeley Madonna software. (DOCX) [file pcbi.1004598.s001.docx]

**Supporting Information.**

**S1 Text. Supplemental Methods.**

**MATLAB routines for the calculation of Gβγ and Gα available for GIRK with the graded contribution model**

Template for the calculation of Gβγ_total_

[c0,c1,c2,c3,c4, cf,gbg,gbgt]=

solve ('4*c0*gbg= K_D_*c1', '3*c1*gbg=2* K_D_ *c2', '2*c2*gbg=3 *K_D_ *c3',

'c3*gbg=4* K_D_ *c4','c0+c1+c2+c3+c4=n*166e-9 ',

'gbg+c1+2*c2+3*c3+4*c4=m',

'.013*c1+.065*c2+.26*c3+c4=cf',

’cf=p /(72836285*.105)')

% Copy the system to worksheet

% Enter the following:

% n – number of channels per μm^2^

% p – current in μA (I_total_)

% gbgt - Gβγ_total_

Template for the calculation of Gα_total_

[c0, c1, c2, c3, c4, cf, ga, gagbg, gat, gbg]=

solve ('c0*gbg= 4*K_D_*c1', '3*c1*gbg=2* K_D_*c2' , '2*c2*gbg=3 *K_D_ *c3',

'c3*gbg=4* K_D_*c4', 'c0+c1+c2+c3+c4=n*166e-9',

'gbg+gagbg+c1+2*c2+3*c3+4*c4=m', ‘ga+gagbg=gat’, ‘0.0013*gagbg=0.7e6*ga*gbg ’

'.013*c1+.065*c2+.26*c3+c4=cf',

’cf=q /(72836285*.105)')

% Copy the system to worksheet

% Enter the following:

% n – number of channels per μm^2^

% q – current in μA (I_basal_)

% m - Gβγ_total_

% gat - Gα_total_

Template for the simulation of a Gβγ overexpression experiment

[c0, c1, c2, c3, c4, cf, curr, ga, gagbg, gat, gbg]=

solve ('c0*gbg= 4*K_D_*c1', '3*c1*gbg=2* K_D_*c2' , '2*c2*gbg=3 *K_D_ *c3',

'c3*gbg=4* K_D_*c4', 'c0+c1+c2+c3+c4=n*166e-9',

'gbg+gagbg+c1+2*c2+3*c3+4*c4=m', ‘ga+gagbg=gat’, ‘0.0013*gagbg=0.7e6*ga*gbg ’

'.013*c1+.065*c2+.26*c3+c4=cf',

’curr=cf*(72836285*.105)')

% Copy the system to worksheet

% Enter the following:

% n – number of channels per μm^2^

% curr – current in μA

% m - Gβγ_total_

% gat - Gα_total_

All results are in M

**Calculations of model predictions for a range of parameters using the Berkeley Madonna software.**

To simulate macroscopic GIRK1/2 currents or their ratios (R_a_, R_βγ_) for a range of arguments in order to produce continuous curves (Figs. 5, 7C, S4, S7, S8), we utilized Berkley Madonna software which implements 4^th^ order Runge-Kutta method for numerical solution of differential equations. In these calculations, channel activation process by Gβγ was described by following system of differential equations:

,

where all symbols are defined as in the main text of the paper, and k_1_ and k_-1_ are forward and reverse rate constants of GIRK-Gβγ interaction, respectively. We used k_1_ = 10^7^ M^-1^s^-1^ and k_-1_ = 0.5 s^-1^. These rate constants of Gβγ and GIRK channel interaction are unknown, but these values are in Smoluchowski limit [[1](#_ENREF_1)] and render K_D_ = 50 nM which we used in main calculations of our model. The rate constants of interaction between Gβγ and Gα_i_ were k_off_ = 0.0013 s^-1^ and k_on_ = 0.7×10^6^ M^-1^s^-1^ [[2](#_ENREF_2)].

**Supplemental References**

1. Berg OG, von Hippel PH (1985) Diffusion-controlled macromolecular interactions. Annu Rev Biophys Biophys Chem 14: 131-160.

2. Sarvazyan NA, Remmers AE, Neubig RR (1998) Determinants of G_i1_α and βγ binding. Measuring high affinity interactions in a lipid environment using flow cytometry. J Biol Chem 273: 7934-7940.

3. Axelrod D, Wang MD (1994) Reduction-of-dimensionality kinetics at reaction-limited cell surface receptors. Biophys J 66: 588-600.

4. Kholodenko BN, Hoek JB, Westerhoff HV (2000) Why cytoplasmic signalling proteins should be recruited to cell membranes. Trends Cell Biol 10: 173-178.

5. Shoup D, Lipari G, Szabo A (1981) Diffusion-controlled bimolecular reaction rates. The effect of rotational diffusion and orientation constraints. Biophys J 36: 697-714.

6. Lauffenburger D, Linderman JJ (1996) Receptors: Models for Binding, Trafficking, and Signaling. New York: Oxford University Press.

7. Runnels LW, Scarlata SF (1998) Regulation of the rate and extent of phospholipase Cβ2 effector activation by the βγ subunits of heterotrimeric G proteins. Biochemistry 37: 15563-15574.

8. Runnels LW, Scarlata SF (1999) Determination of the affinities between heterotrimeric G protein subunits and their phospholipase Cβ effectors. Biochemistry 38: 1488-1496.

9. Yakubovich D, Rishal I, Dascal N (2005) Kinetic modeling of Na^+^-induced, Gβγ -dependent activation of G-protein-gated K^+^ channels. J Mol Neurosci 25: 7-20.

10. Silverman SK, Lester HA, Dougherty DA (1996) Subunit stoichiometry of a heteromultimeric G protein-coupled inward- rectifier K^+^ channel. J Biol Chem 271: 30524-30528.

11. Grasser E, Steinecker B, Ahammer H, Schreibmayer W (2008) Subunit stoichiometry of heterologously expressed G-protein activated inwardly rectifying potassium channels analysed by fluorescence intensity ratio measurement. Pflugers Arch 455: 1017-1024.

# Supporting Figure Legends

**S1 Fig. Block of inward currents in cultured hippocampal neurons by TPNQ and Ba^2+^. (A)** Ba^2+^ (1 mM) blocks a greater fraction of the total inward current in high-K^+^ solution, compared to TPNQ (120 nM). The experimental protocol was the same as in Fig 1, with the additional step of Ba^2+^ addition after TPNQ. ΔTPN and ΔBa denote the magnitudes (shown by double-speared arrows) of TPNQ- and Ba-blocked currents, respectively. Note that Ba^2+^ blocked a much greater fraction of the total inward current in high-K^+^ solution, most probably of the block of additional Ba^2+^ -sensitive channels present in these neurons. **(B)** Comparison of average TPNQ- and Ba^2+^-blocked currents in 14 cells of one batch of neurons. Statistical significance (p<0.001) was determined using Wilcoxon Signed Rank test (the data did not pass normality test).

**S2 Fig. GIRK1/2 currents in oocytes.** Holding potential was -80 mV, low-K^+^ and high-K^+^ solutions contained 2 and 24 mM K^+^, respectively (K^+^ was replaced for Na^+^). Net GIRK currents were determined by subtracting the current remaining after the addition of 5 mM BaCl_2_. (**A**) I_basal_ and I_evoked_ in an oocyte expressing m2R, GIRK1 and GIRK2. Calculation of R_a_ was done in every cell from its own I_basal_ and I_evoked_. (**B**) I_βγ_ in an oocyte expressing m2R, GIRK1, GIRK2 and Gβγ. Note that adding ACh did not evoke a significant additional GIRK current, suggesting full activation by Gβγ. R_βγ_ was calculated in each cell by dividing its own I_βγ_ by the average I_βγ_ from the control group of the same experiment in which no Gβγ was coexpressed. **(**C**)** Expression of m2R in a wide range of doses does not affect I_basal_. 5-8 oocytes have been tested in each group. There were no significant differences between treatments as tested by one-way ANOVA.

**S3 Fig. Characterization of YFP-labeled GIRK1 and Gβ. (A, B)** Single channel parameters of GIRK1/2 and YFP-GIRK1/2 channels are very similar. **(A)** Cell-attached records of channel activity expressing the channel and Gβγ (5 ng RNA). **(B)** Comparison of average i­_single_  and P_o_. Data are from oocytes of the same batch, recorded during a two-day experiment. **(C, D)** The anti-Gβ antibody similarly recognizes YFP-labeled bovine and *Xenopus* Gβ subunits in Western blots of manually pealed plasma membranes. Data are from 4 separate experiments. For Western blots, 15 to 20 plasma membranes were pooled. For confocal imaging, groups of 3-16 oocytes were examined, and the average fluorescence level was compared with that of YFP-GIRK1/2 (therefore the statistical significance was calculated using paired t-test). The density of the latter was calculated from the measurement of currents as explained in the text. In each experiment, both confocal imaging, current measurement and Western blots of manually peeled membranes were done in oocytes of the same donor. There was a good agreement for surface density estimates of YFP-Gβ-XL from confocal "molecular ruler" measurements and from quantitative Western blots, either in absolute terms as molecules/µm^2^ (**C**) or in relative terms, normalized to estimates of YFP-Gβ in each experiment (**D**). YFP fluorescence can be safely assumed to be independent of the species of fused Gβ (mammalian or *Xenopus*). Therefore, similar estimates of surface density observed from confocal imaging and Western blots suggest that the Gβ antibody used here recognizes the oocyte's endogenous Gβ in Western blots similarly to the coexpressed mammalian (bovine) Gβ_1_.

**S4 Fig**. **Simulation of density-dependent changes in whole-cell GIRK1/2 activity.** Experimental data (from Table 1) are shown as red circles (mean ± SEM). The simulations of currents and Ra were done using the graded contribution model. **(A)** Testing the hypothesis that the endogenous Gαβγ heterotrimers are the only source of Gβγ for GIRK activation; I_basal_ is due to spontaneous dissociation of Gαβγ into Gα^GDP^ and Gβγ (see Fig. 2A). Simulations were performed assuming that only part (1 or 10 molecules/µm^2^, black and red curves) or all (24 molecules/µm^2^, blue curves) endogenous G proteins can donate Gβγ to activate GIRK1/2. Note that no satisfactory description of data can be obtained under any of these conditions. The simulated I_basal­_ is too low; for high channel densities, also the full I_evoked_ could not be obtained even assuming that all endogenous Gαβγ (i.e. all 24 molecules/µm^2^) could release Gβγ and activate GIRK. **(B)** Testing the hypothesis that the expressed GIRK1/2 recruits additional endogenous G protein subunits to the PM, e.g. from other cellular compartments. Simulations were done assuming that each GIRK1/2 channel recruits from 1 to 4 G_i/o_ heterotrimers. The recruited Gα and Gβγ were added to the pre-existing endogenous plasma membrane-attached Gαβγ before Gβγ expression. **(C)** Testing the hypothesis that the expressed GIRK1/2 recruits additional endogenous Gβγ, but not Gα, to the PM; the rest was done as in B. Calculations in (B) and (C) assumed 24 molecules/µm^2^ of endogenous G_i/o_ available for GIRK. Similar results were obtained assuming 10 molecules/µm^2^ (data not shown). Simulations as in A-C were also done with the concerted model, yielding similar results (data not shown).

**S5 Fig. The concerted activation model supports the unequal stoichiometry estimates of Gβγ and Gα available for GIRK1/2.** The plots present the calculated amounts of Gβγ and Gα available for GIRK1/2 using the concerted model for a range of K_D_ for the GIRK-Gβγ interaction (5-100 nM), for the three channel density groups of Table 1.

**S6 Fig.** **The presence of Gβγ-independent intrinsic activity and the dimensions of submembrane reaction space do not significantly alter the estimates of GIRK1/2-available G proteins subunits.** Calculations were done assuming K_D_=50 nM for the GIRK-Gβγ interaction. **(A-C),** the impact of Gβγ-independent basal activity. Calculation were done for Gβγ-independent intrinsic activity of a single channel ranging from 1% to 10% of P_o,max_. Available Gβγ (**A**), Gα (**B**) and the Gβγ-independent fraction of I_basal_ (**C**) were calculated for the three channel density groups of Table 1. **(D, E)** Varying the submembrane space thickness in a wide range, 1-20 nm, does not significantly change the estimates of functional stoichiometry of GIRK1/2-Gβγ-Gα.

**S7 Fig. Simulations of the Gβγ dose-response experiment for a range of assumed Gβγ densities.** Because in the experiment of Fig. 7 the actual density of Gβγ in the PM has not been directly measured, the calculations of Fig. 7C assumed that it was equal to the average density of 30 Gβγ molecules/µm^2^ (with 5 ng RNA), as measured in other 4 experiments done during the same time period. Here, we run simulations as in Fig. 7C for 20 or 44 molecules Gβγ/µm^2^ (**A, C**) and compare the result with that of Fig. 7C (shown here again in **B** for a direct comparison). The color codes are as in Fig. 7: the blue line presents the simulation using graded contribution model and amounts of Gα and Gβγ (without coexpressed Gβγ) calculated as explained in Fig. 7 legend, and red, black and green lines show simulation with endogenous G proteins only and no Gβγ recruitment allowed.

**S8 Fig. Another experiment on dose-dependent activation of GIRK1/2 by coexpressed Gβγ.** The presentation in similar to that of Fig. 7. Gβ was coexpressed with Gγ-YFP in incremental doses, and with a constant amount (1 ng RNA) of wt GIRK1/2. RNA of Gγ-YFP was always half of that of Gβ RNA, by weight. **(A)** Gβγ-YFP fluorescence levels (grey bars, left Y-axis) and GIRK currents (red circles, right Y-axis) are shown on the same plot. GIRK1/2 density, calculated from I_βγ_ of the 17 ng Gβγ-YFP group, was 13 molecules/μm^2^. In addition, we injected YFP-GIRK1/GIRK2 (5 ng GIRK1-YFP) and measured I_basal_ which was 8.4± 1.1 µA (n=11), comparable to I_basal_ of unlabeled GIRK1/2 (9.4±0.8 µA). Thus, we assumed the same density of ~13 channels/µm^2^ for labeled and unlabeled channels. Since the YFP-GIRK1/2 gave a fluorescent signal of 1237 ±221 AU (n=7), this signal was assumed to correspond to 26 YFP molecules/µm^2^. This number was used as the basis of calculations of Gβγ-YFP density for plots shown in B. **(B)** Comparison of measured I_βγ_ or R_βγ_ (red circles) and simulated currents or R­_βγ_ (blue curves). The left and right Y-axes are related to I_βγ_ and R_βγ_, respectively. Available Gα and Gβγ (before Gβγ coexpression) were estimated from I_total_ and I_basal_, giving 3.82 and 0.42 molecules/µm^2^ of Gβγ and Gα, respectively.

**S9 Fig. Estimated stoichiometries of Gα and Gβγ available for GIRK in neurons and oocytes in a range of I_βγ_/I_total_ ratios and P_o,max_.** Whereas for the oocytes the actual I_βγ_/I_total_ ratio and P_o,max_ are known, in neurons these parameters are not known. Both parameters affect the calculated channel density and could affect the estimates of stoichiometry. The calculations shown in this Figure demonstrate the same general trend in stoichiometries of GIRK1/2, Gβγ and Gα as we have found in the previous analysis in the oocytes, in a range of I_βγ_/I_total_ ratios (for neurons and oocytes; **A** and **B**) and P_o,max_ (for neurons; **C**). The estimates of Gβγ are around 3-4/channel and relatively independent of I_basal_, and those of Gα are below 2 and drop sharply with the increase in I_basal_. Generally, the lowest channel density is most sensitive to perturbations, and, for the lowest simulated I_βγ_/I_total_ ratio, calculated Gβγ/channel and Gα/channel exceed our usual estimates.
