# Supplementary material for: A Quantitative Model of the GIRK1/2 Channel Reveals That Its Basal and Evoked Activities Are Controlled by Unequal Stoichiometry of Gα and Gβγ
Source: PLoS Comput Biol. 2015 Nov 6;11(11):e1004598. doi: 10.1371/journal.pcbi.1004598 (PMC4636287; doi:10.1371/journal.pcbi.1004598)
Supplement: S2 Text — Conversion from channel densities to concentrations; GIRK1/2 stoichiometry; Estimating steady-state open probability with the “separate gating transitions” model. (DOCX) [file pcbi.1004598.s002.docx]

**S2 Text. Supplemental discussion**

**Conversion from channel densities to concentrations.**

There is no consensus about the way the reaction rates are changed by the reduction of dimensionality [[3](#_ENREF_3),[4](#_ENREF_4),[5](#_ENREF_5)]. Nevertheless, for proteins that interact in the submembrane cytosolic space conversion from two-dimensional rate constants and densities into three-dimensional rate constants and concentrations is a well-accepted paradigm. This is particularly useful, and has been used in the past, in calculations involving GPCRs and G proteins or effectors and G proteins (e.g. [[6](#_ENREF_6),[7](#_ENREF_7),[8](#_ENREF_8),[9](#_ENREF_9)]).

**GIRK1/2 stoichiometry.**

Silverman et al. [[10](#_ENREF_10)] have shown that GIRK1/4 channels with 2:2 stoichiometry are preferentially expressed in oocytes and contribute the majority of the macroscopic current, but 3:1 or 1:3 stoichiometry is viable (see also [[11](#_ENREF_11)]). No such data are available for GIRK1/2. Generally speaking, for macroscopic currents, the exact subunit stoichiometry does not matter, because for a constant RNA ratio as used here (equal amounts of RNAs for both subunits), a relatively constant composition of channel population (in terms of stoichiometry) is expected, subject to natural variability. We cannot exclude the possibility that, in some single-channel recordings, we observed channels of unequal stoichiometry. Nevertheless, since simulations are based on average isingle and Po,max­ from a rather large sample of patches (since the closing of data collection for this paper, we have recorded more single GIRK1/2 channels and always obtain a similar Po,max around 0.11; data not shown), we posit that the average values of Po,max and isingle faithfully represent the population of channels seen in our macroscopic recordings.

**Estimating steady-state open probability with the “separate gating transitions” model.**

For clarity, here we present the separate gating transitions model from Fig. 2B using the notation which will be utilized in the following equations.

$$\begin{matrix} C_{0}^{c} \\ ⇕ \\ C_{0}^{o} \end{matrix}\begin{matrix} \Leftrightarrow& C_{1}^{c} & \Leftrightarrow\\ & ⇕ & \\ \Leftrightarrow& C_{1}^{o} & \Leftrightarrow\end{matrix}\begin{matrix} C_{2}^{c} & \Leftrightarrow& C_{3}^{c} \\ ⇕ & & ⇕ \\ C_{2}^{o} & \Leftrightarrow& C_{3}^{o} \end{matrix}\begin{matrix} \Leftrightarrow& C_{4}^{c} & \\ & ⇕ & \\ \Leftrightarrow& C_{4}^{o} & \end{matrix}$$

where $C_{x}^{c}$ denotes concentration of channels occupied by x Gβγ molecules in closed state and $C_{x}^{o}$ is is the concentration of channels occupied by x Gβγ molecules in open state.

The open probability for the above scheme can be calculated according to:

(S1)

The equilibrium constant Kx between $C_{x}^{c}$ and $C_{x}^{o}$ is

Kx=$C_{x}^{o}$/$C_{x}^{c}$ (S2)

and Po,x is the open probability of x Gβγ molecules occupied channel and can be defined as

(S3)

If Cx is the concentration of channels occupied by x Gβγ molecules, then

Cx = $C_{x}^{c}$+ $C_{x}^{o}$ (S4)

and if Ctotal is total channel concentration, then

(S5)

Thus (S1) can be rearranged

(S6)

Solving (S2) and (S4) for $C_{x}^{o}$ renders

(S7)

Substituting (S3) into (S7) renders

(S8)

Substituting (S8) into (S6) renders

(S9)

If fp,x is the fraction of Po,max (maximal observable Po) and is defined as fp,x = Po,x/Po.max and *x* is the fraction of channels occupied by x Gβγ molecules (defined as *x*= Cx/Ctotal), then substitution of definitions of fp,x and *x* to (S9) will render

(S10)

This equation is identical to Equation 6 (Methods).

The graded contribution model and the more elaborated independent gating transitions model will predict distinct single channel behavior of GIRK1/2, which may need to be investigated in detail to distinguish between the two models. Nevertheless, as shown above, the description of steady-state Po by the two models converges to Equation 6 (Methods), suggesting that this is a general equation which can describe GIRK1/2 macroscopic gating for any number of open states. As a consequence, the two models should produce identical fractional macroscopic steady-state currents (Po/Po,max, where Po,max is equivalent to I/Iβγ, where I is either Ibasal, Itotal or Ievoked) once the channel is occupied by x Gβγ. In other words, for steady state description of macroscopic currents that we present in our study, the results are expected to be the similar both for the graded contribution model as well as for the more elaborated independent gating transitions model.

**Supplemental References**

1. Berg OG, von Hippel PH (1985) Diffusion-controlled macromolecular interactions. Annu Rev Biophys Biophys Chem 14: 131-160.

2. Sarvazyan NA, Remmers AE, Neubig RR (1998) Determinants of Gi1a and bg binding. Measuring high affinity interactions in a lipid environment using flow cytometry. J Biol Chem 273: 7934-7940.

3. Axelrod D, Wang MD (1994) Reduction-of-dimensionality kinetics at reaction-limited cell surface receptors. Biophys J 66: 588-600.

4. Kholodenko BN, Hoek JB, Westerhoff HV (2000) Why cytoplasmic signalling proteins should be recruited to cell membranes. Trends Cell Biol 10: 173-178.

5. Shoup D, Lipari G, Szabo A (1981) Diffusion-controlled bimolecular reaction rates. The effect of rotational diffusion and orientation constraints. Biophys J 36: 697-714.

6. Lauffenburger D, Linderman JJ (1996) Receptors: Models for Binding, Trafficking, and Signaling. New York: Oxford University Press.

7. Runnels LW, Scarlata SF (1998) Regulation of the rate and extent of phospholipase Cb2 effector activation by the bg subunits of heterotrimeric G proteins. Biochemistry 37: 15563-15574.

8. Runnels LW, Scarlata SF (1999) Determination of the affinities between heterotrimeric G protein subunits and their phospholipase Cb effectors. Biochemistry 38: 1488-1496.

9. Yakubovich D, Rishal I, Dascal N (2005) Kinetic modeling of Na+-induced, Gbg -dependent activation of G-protein-gated K+ channels. J Mol Neurosci 25: 7-20.

10. Silverman SK, Lester HA, Dougherty DA (1996) Subunit stoichiometry of a heteromultimeric G protein-coupled inward- rectifier K+ channel. J Biol Chem 271: 30524-30528.

11. Grasser E, Steinecker B, Ahammer H, Schreibmayer W (2008) Subunit stoichiometry of heterologously expressed G-protein activated inwardly rectifying potassium channels analysed by fluorescence intensity ratio measurement. Pflugers Arch 455: 1017-1024.
