# Supplementary material for: A Quantitative Model of the GIRK1/2 Channel Reveals That Its Basal and Evoked Activities Are Controlled by Unequal Stoichiometry of Gα and Gβγ
Source: PLoS Comput Biol. 2015 Nov 6;11(11):e1004598. doi: 10.1371/journal.pcbi.1004598 (PMC4636287; doi:10.1371/journal.pcbi.1004598)
Supplement: S1 Table — Data are from 2 to 4 experiments, for each group, shown as mean ± SEM. We did not include experiments with extremely large Gαi3 RNA quantities, as expression of higher doses of Gαi3 usually reduced Itotal, indicating a general Gβγ scavenging effect rather than priming. (DOCX) [file pcbi.1004598.s003.docx]

**Supplemental Table 1**

**S1 Table.** Effect of coexpression of Gα_i3_ on GIRK1/2 currents in oocytes. Data are from 2 to 4 experiments, for each group, shown as mean ± SEM. We did not include experiments with extremely large Gα_i3_ RNA quantities, as expression of higher doses of Gα_i3_ usually reduced I_total_, indicating a general Gβγ scavenging effect rather than priming.

| **50-200 pg GIRK1/2 (n=10)** | | | | **50-200 pg GIRK1/2+0.5-2 ng Gαi3 (n=10)** | | | |
| --- | --- | --- | --- | --- | --- | --- | --- |
| I_basal_ (µA) | I_evoked_ (µA) | I_total_ (µA) | R_a_ | I_basal_ (µA) | I_evoked_ (µA) | I_total_ (µA) | R_a_ |
| 4.8±0.42 | 4.2±0.76 | 9.1±1 | 1.9±0.14 | 1.2±0.2 | 6.8±0.9 | 8.0±0.7 | 10.5±2.7 |
|  |  |  |  |  |  |  |  |
| **0.5-1 ng GIRK1/2 (n=27)** | | | | **0.5-1 ng GIRK1/2 + 5-10 ng Gαi3 (n=16)** | | | |
| I_basal_ (µA) | I_evoked_ (µA) | I_total_ (µA) | R_a_ | I_basal_ (µA) | I_evoked_ (µA) | I_total_ (µA) | R_a_ |
| 12.6±0.7 | 2.5±0.3 | 15.1±0.8 | 1.2±0.03 | 2.4±0.6 | 11.5±0.8 | 13.9±1.2 | 10.4±1.7 |
