# Supplementary figures and images for: A Quantitative Model of the GIRK1/2 Channel Reveals That Its Basal and Evoked Activities Are Controlled by Unequal Stoichiometry of Gα and Gβγ
Source: PLoS Comput Biol. 2015 Nov 6;11(11):e1004598. doi: 10.1371/journal.pcbi.1004598 (PMC4636287; doi:10.1371/journal.pcbi.1004598)

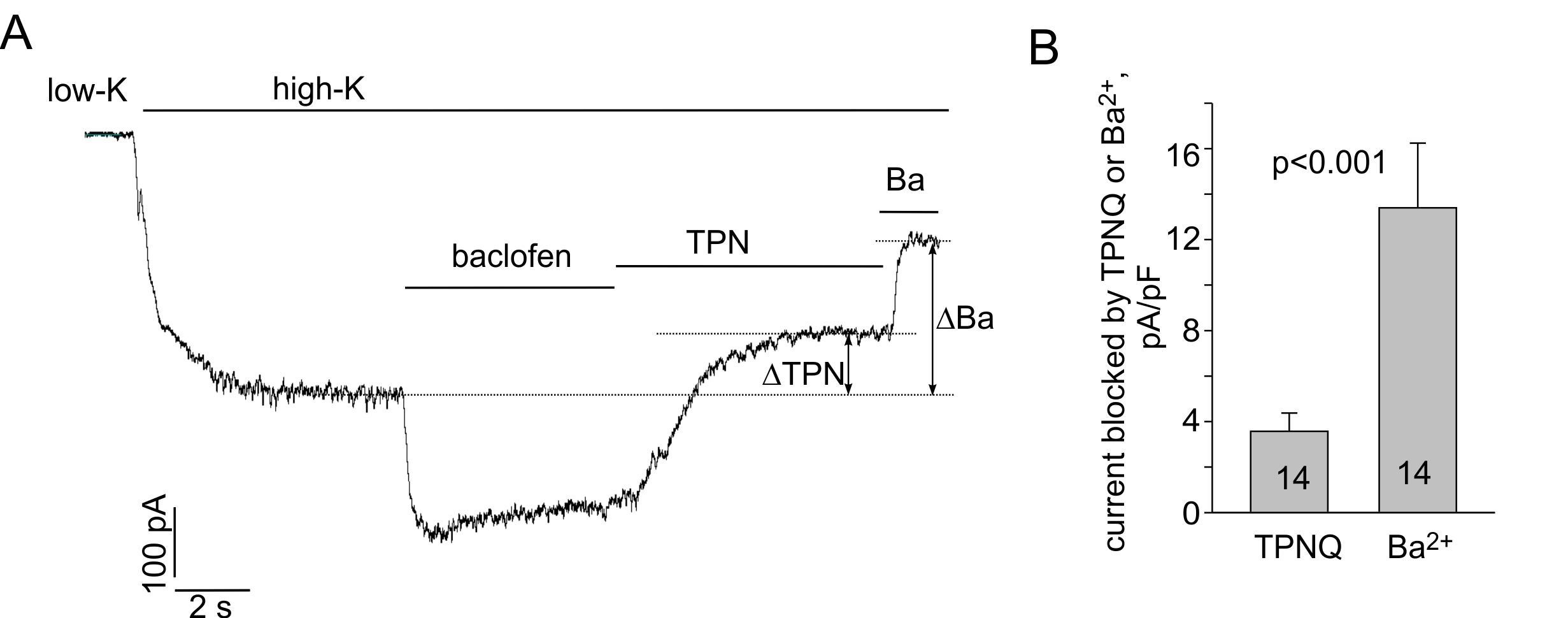

Supplement: S1 Fig — (A) Ba2+ (1 mM) blocks a greater fraction of the total inward current in high-K+ solution, compared to TPNQ (120 nM). The experimental protocol was the same as in Fig 1, with the additional step of Ba2+ addition after TPNQ. ΔTPN and ΔBa denote the magnitudes (shown by double-speared arrows) of TPNQ- and Ba-blocked currents, respectively. Note that Ba2+ blocked a much greater fraction of the total inward current in high-K+ solution, most probably of the block of additional Ba2+ -sensitive channels present in these neurons. (B) Comparison of average TPNQ- and Ba2+-blocked currents in 14 cells of one batch of neurons. Statistical significance (p<0.001) was determined using Wilcoxon Signed Rank test (the data did not pass normality test). (TIF) [file pcbi.1004598.s004.tif]

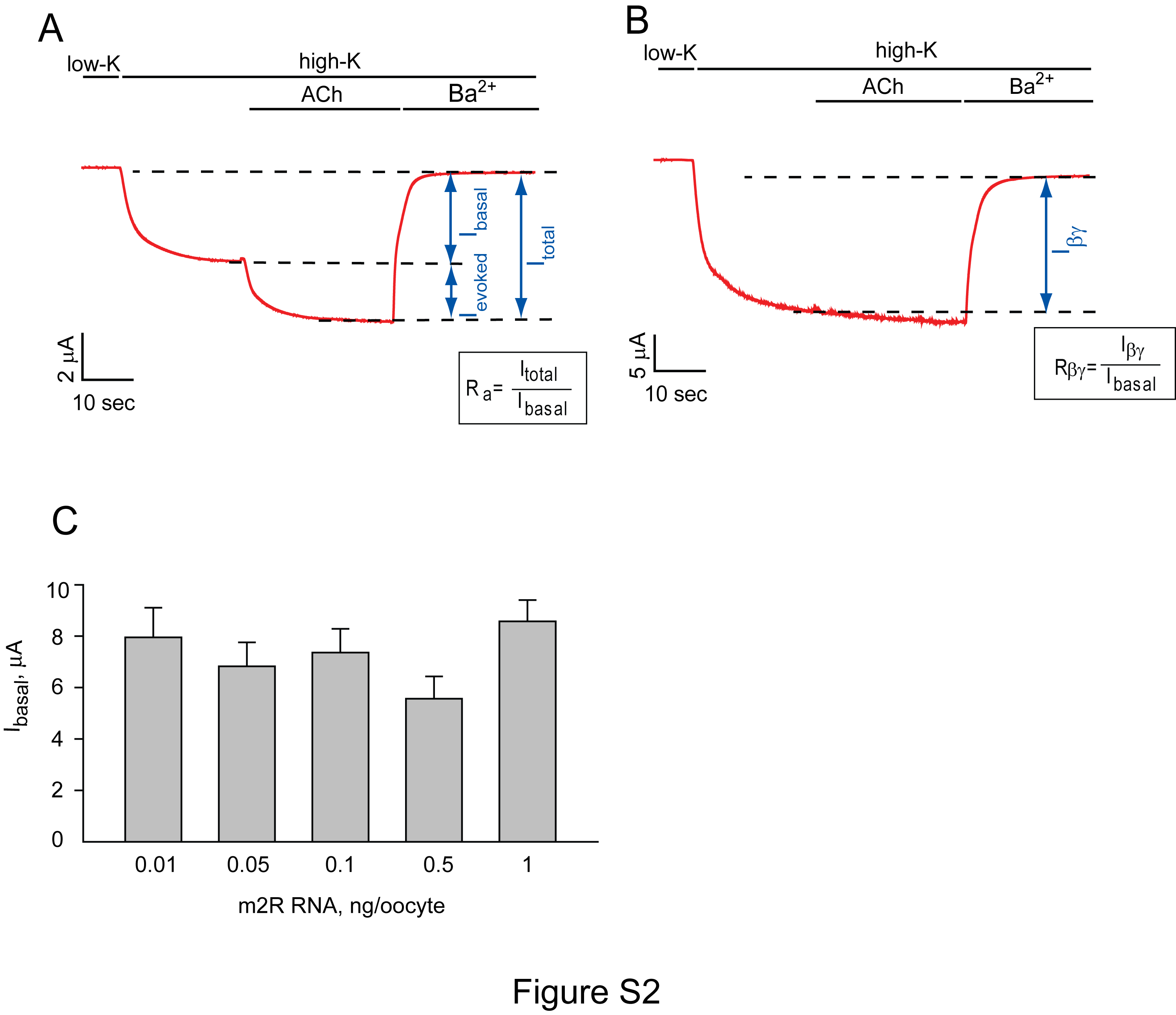

Supplement: S2 Fig — Holding potential was -80 mV, low-K+ and high-K+ solutions contained 2 and 24 mM K+, respectively (K+ was replaced for Na+). Net GIRK currents were determined by subtracting the current remaining after the addition of 5 mM BaCl2. (A) Ibasal and Ievoked in an oocyte expressing m2R, GIRK1 and GIRK2. Calculation of Ra was done in every cell from its own Ibasal and Ievoked. (B) Iβγ in an oocyte expressing m2R, GIRK1, GIRK2 and Gβγ. Note that adding ACh did not evoke a significant additional GIRK current, suggesting full activation by Gβγ. Rβγ was calculated in each cell by dividing its own Iβγ by the average Iβγ from the control group of the same experiment in which no Gβγ was coexpressed. (C) Expression of m2R in a wide range of doses does not affect Ibasal. 5–8 oocytes have been tested in each group. There were no significant differences between treatments as tested by one-way ANOVA. (TIF) [file pcbi.1004598.s005.tif]

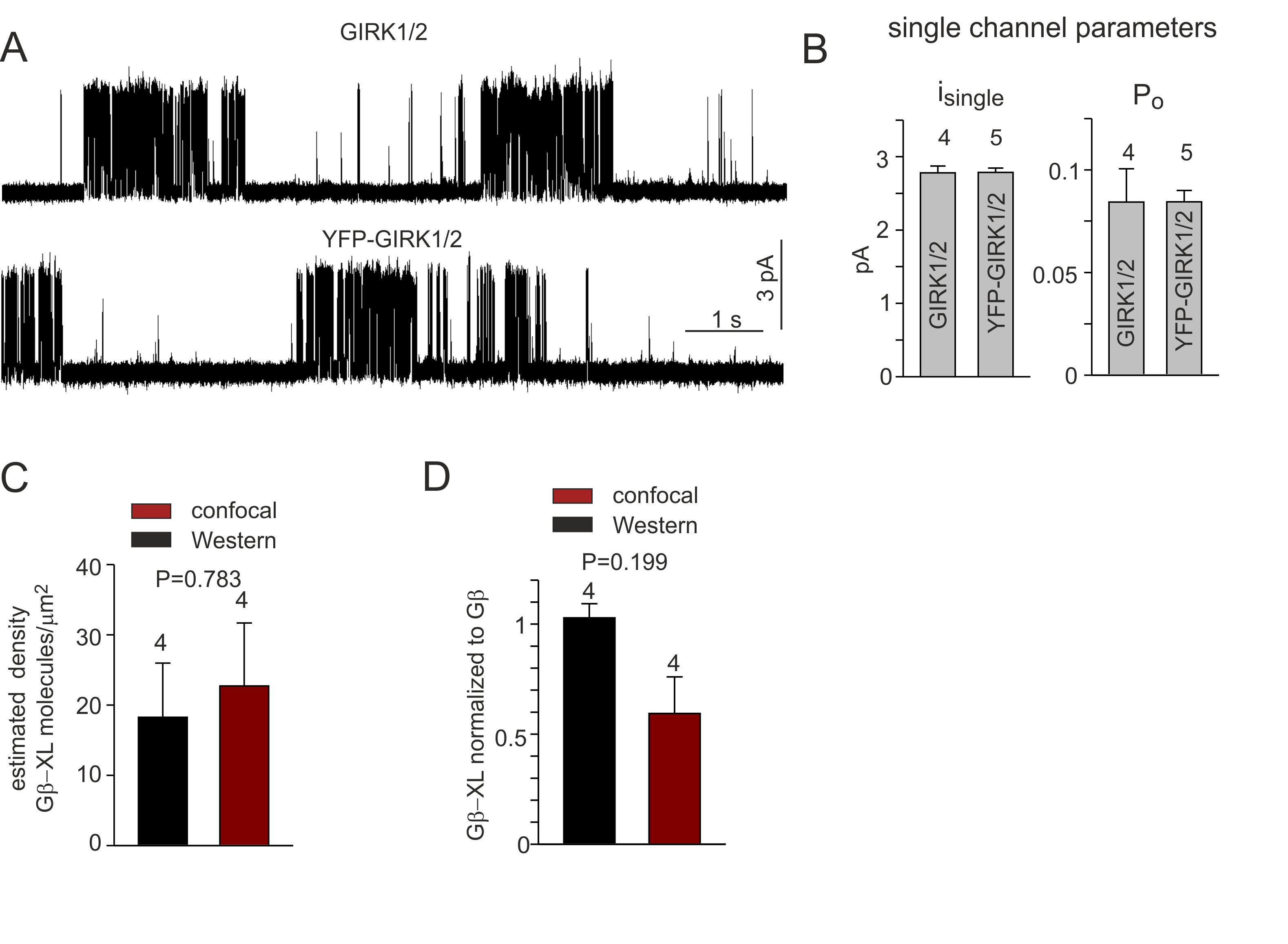

Supplement: S3 Fig — (A, B) Single channel parameters of GIRK1/2 and YFP-GIRK1/2 channels are very similar. (A) Cell-attached records of channel activity expressing the channel and Gβγ (5 ng RNA). (B) Comparison of average isingle and Po. Data are from oocytes of the same batch, recorded during a two-day experiment. (C, D) The anti-Gβ antibody similarly recognizes YFP-labeled bovine and Xenopus Gβ subunits in Western blots of manually pealed plasma membranes. Data are from 4 separate experiments. For Western blots, 15 to 20 plasma membranes were pooled. For confocal imaging, groups of 3–16 oocytes were examined, and the average fluorescence level was compared with that of YFP-GIRK1/2 (therefore the statistical significance was calculated using paired t-test). The density of the latter was calculated from the measurement of currents as explained in the text. In each experiment, both confocal imaging, current measurement and Western blots of manually peeled membranes were done in oocytes of the same donor. There was a good agreement for surface density estimates of YFP-Gβ-XL from confocal "molecular ruler" measurements and from quantitative Western blots, either in absolute terms as molecules/μm2 (C) or in relative terms, normalized to estimates of YFP-Gβ in each experiment (D). YFP fluorescence can be safely assumed to be independent of the species of fused Gβ (mammalian or Xenopus). Therefore, similar estimates of surface density observed from confocal imaging and Western blots suggest that the Gβ antibody used here recognizes the oocyte's endogenous Gβ in Western blots similarly to the coexpressed mammalian (bovine) Gβ1. (TIF) [file pcbi.1004598.s006.tif]

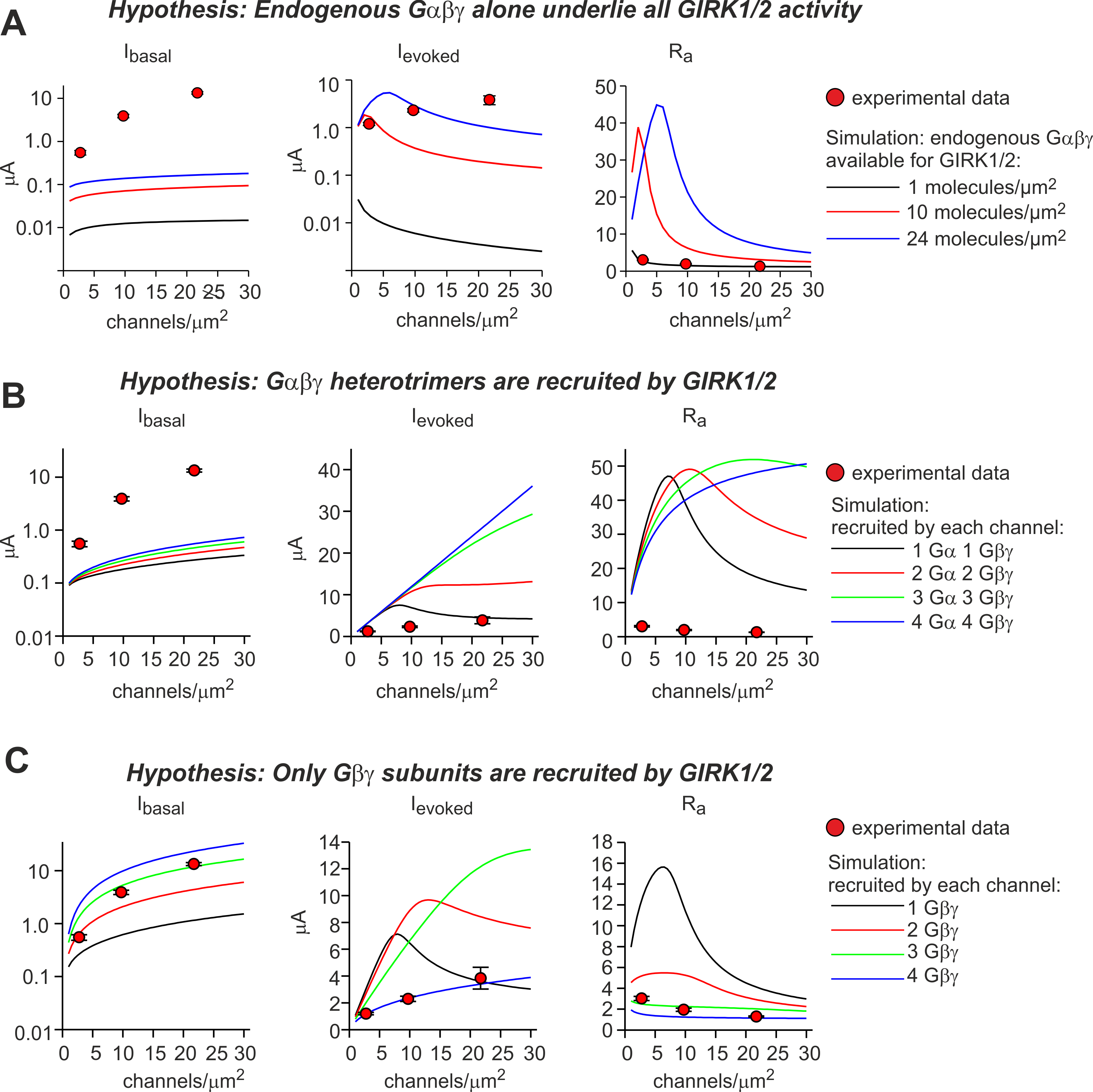

Supplement: S4 Fig — Experimental data (from Table 1) are shown as red circles (mean ± SEM). The simulations of currents and Ra were done using the graded contribution model. (A) Testing the hypothesis that the endogenous Gαβγ heterotrimers are the only source of Gβγ for GIRK activation; Ibasal is due to spontaneous dissociation of Gαβγ into GαGDP and Gβγ (see Fig 2A). Simulations were performed assuming that only part (1 or 10 molecules/μm2, black and red curves) or all (24 molecules/μm2, blue curves) endogenous G proteins can donate Gβγ to activate GIRK1/2. Note that no satisfactory description of data can be obtained under any of these conditions. The simulated Ibasal is too low; for high channel densities, also the full Ievoked could not be obtained even assuming that all endogenous Gαβγ (i.e. all 24 molecules/μm2) could release Gβγ and activate GIRK. (B) Testing the hypothesis that the expressed GIRK1/2 recruits additional endogenous G protein subunits to the PM, e.g. from other cellular compartments. Simulations were done assuming that each GIRK1/2 channel recruits from 1 to 4 Gi/o heterotrimers. The recruited Gα and Gβγ were added to the pre-existing endogenous plasma membrane-attached Gαβγ before Gβγ expression. (C) Testing the hypothesis that the expressed GIRK1/2 recruits additional endogenous Gβγ, but not Gα, to the PM; the rest was done as in B. Calculations in (B) and (C) assumed 24 molecules/μm2 of endogenous Gi/o available for GIRK. Similar results were obtained assuming 10 molecules/μm2 (data not shown). Simulations as in A-C were also done with the concerted model, yielding similar results (data not shown). (TIF) [file pcbi.1004598.s007.tif]

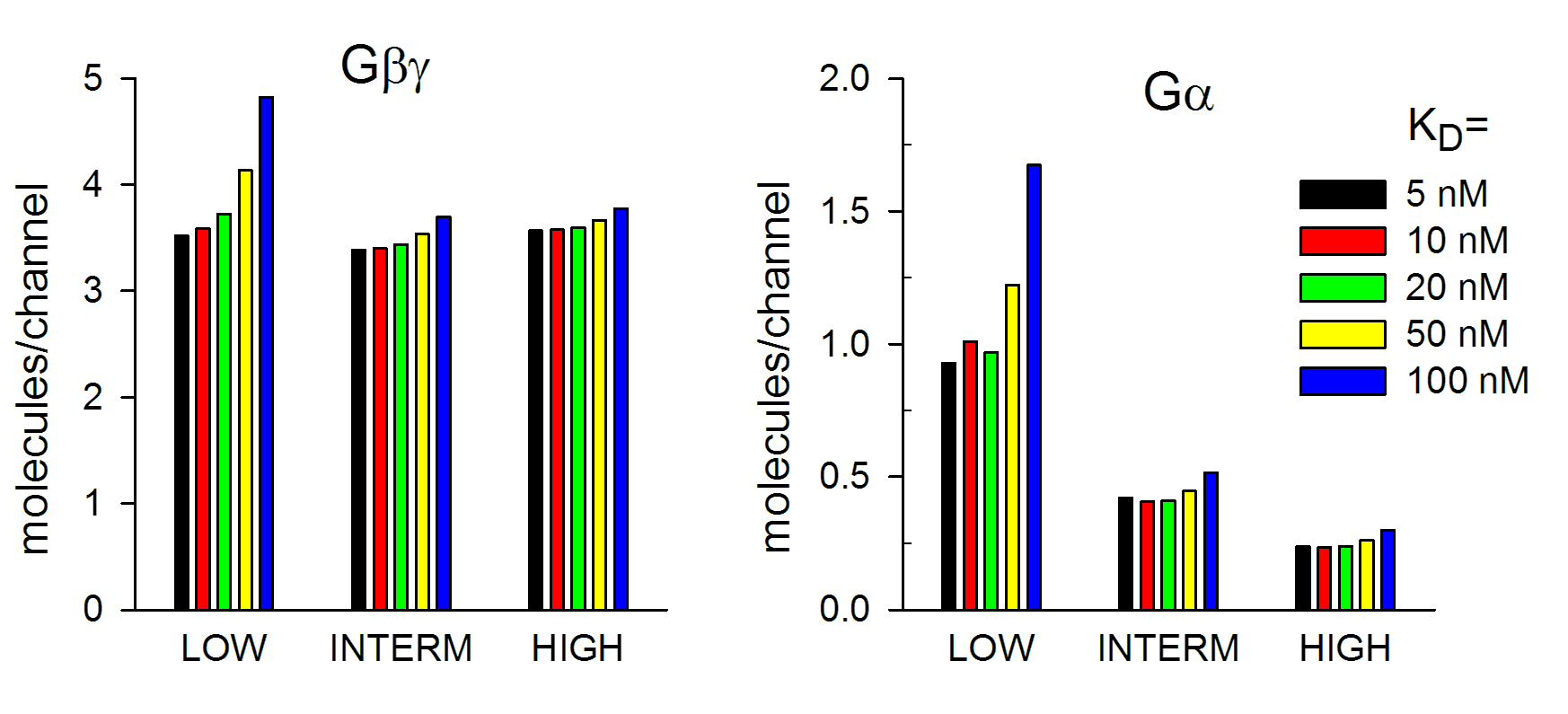

Supplement: S5 Fig — The plots present the calculated amounts of Gβγ and Gα available for GIRK1/2 using the concerted model for a range of KD for the GIRK-Gβγ interaction (5–100 nM), for the three channel density groups of Table 1. (TIF) [file pcbi.1004598.s008.tif]

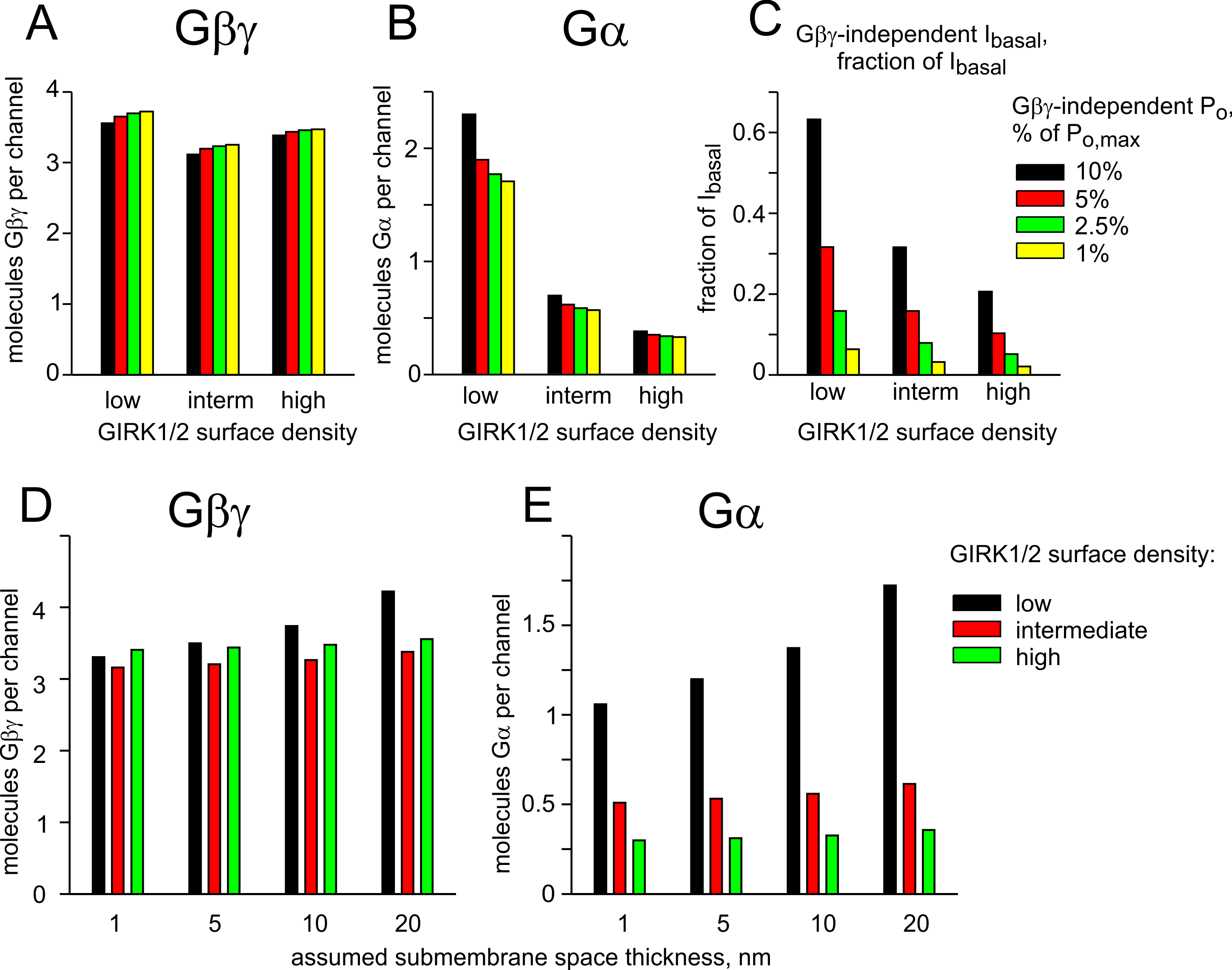

Supplement: S6 Fig — Calculations were done assuming KD = 50 nM for the GIRK-Gβγ interaction. (A-C), the impact of Gβγ-independent basal activity. Calculation were done for Gβγ-independent intrinsic activity of a single channel ranging from 1% to 10% of Po,max. Available Gβγ (A), Gα (B) and the Gβγ-independent fraction of Ibasal (C) were calculated for the three channel density groups of Table 1. (D, E) Varying the submembrane space thickness in a wide range, 1–20 nm, does not significantly change the estimates of functional stoichiometry of GIRK1/2-Gβγ-Gα. (TIF) [file pcbi.1004598.s009.tif]

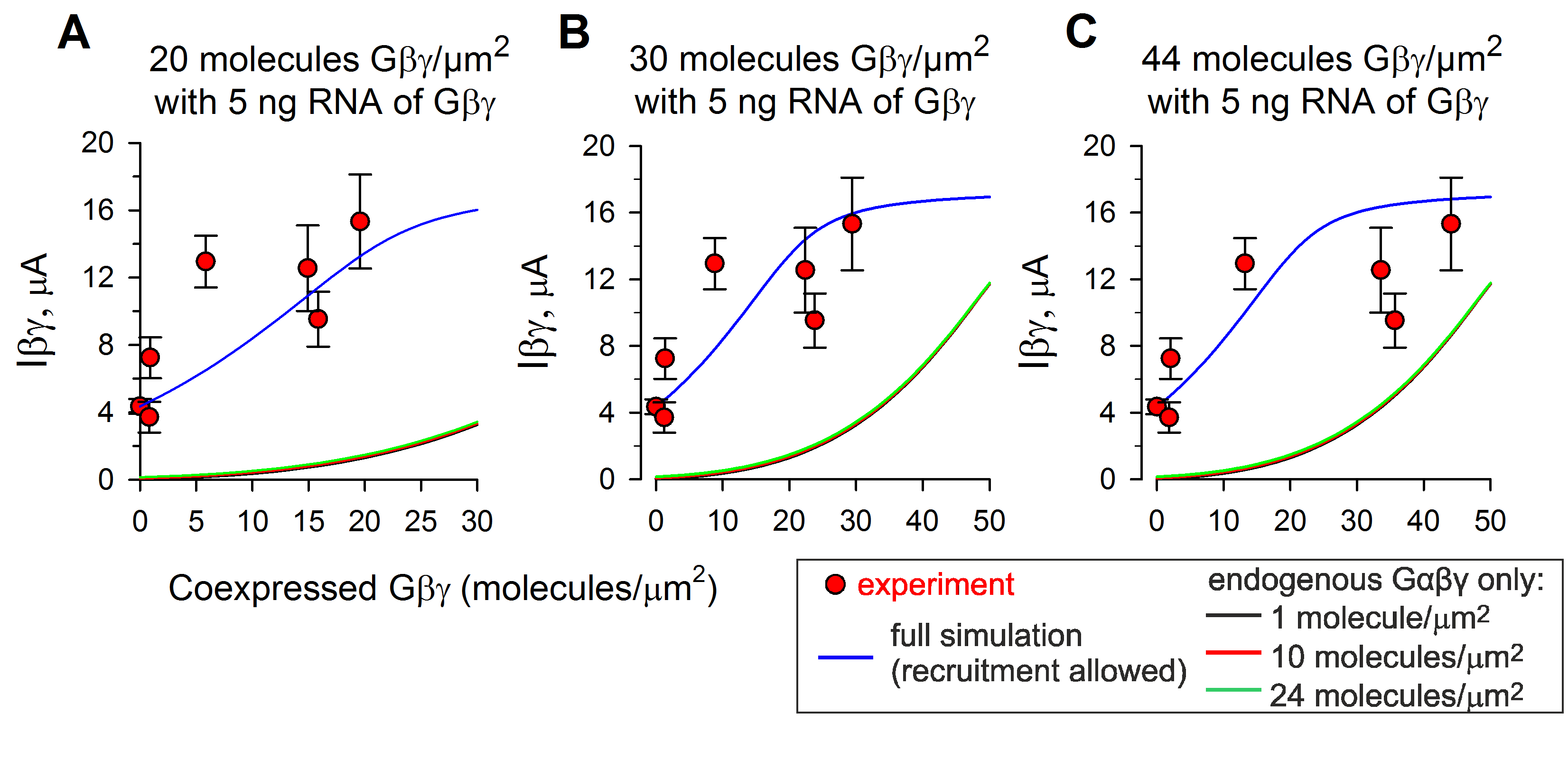

Supplement: S7 Fig — Because in the experiment of Fig 7 the actual density of Gβγ in the PM has not been directly measured, the calculations of Fig 7C assumed that it was equal to the average density of 30 Gβγ molecules/μm2 (with 5 ng RNA), as measured in other 4 experiments done during the same time period. Here, we run simulations as in Fig 7C for 20 or 44 molecules Gβγ/μm2 (A, C) and compare the result with that of Fig 7C (shown here again in B for a direct comparison). The color codes are as in Fig 7: the blue line presents the simulation using graded contribution model and amounts of Gα and Gβγ (without coexpressed Gβγ) calculated as explained in Fig 7 legend, and red, black and green lines show simulation with endogenous G proteins only and no Gβγ recruitment allowed. (TIF) [file pcbi.1004598.s010.tif]

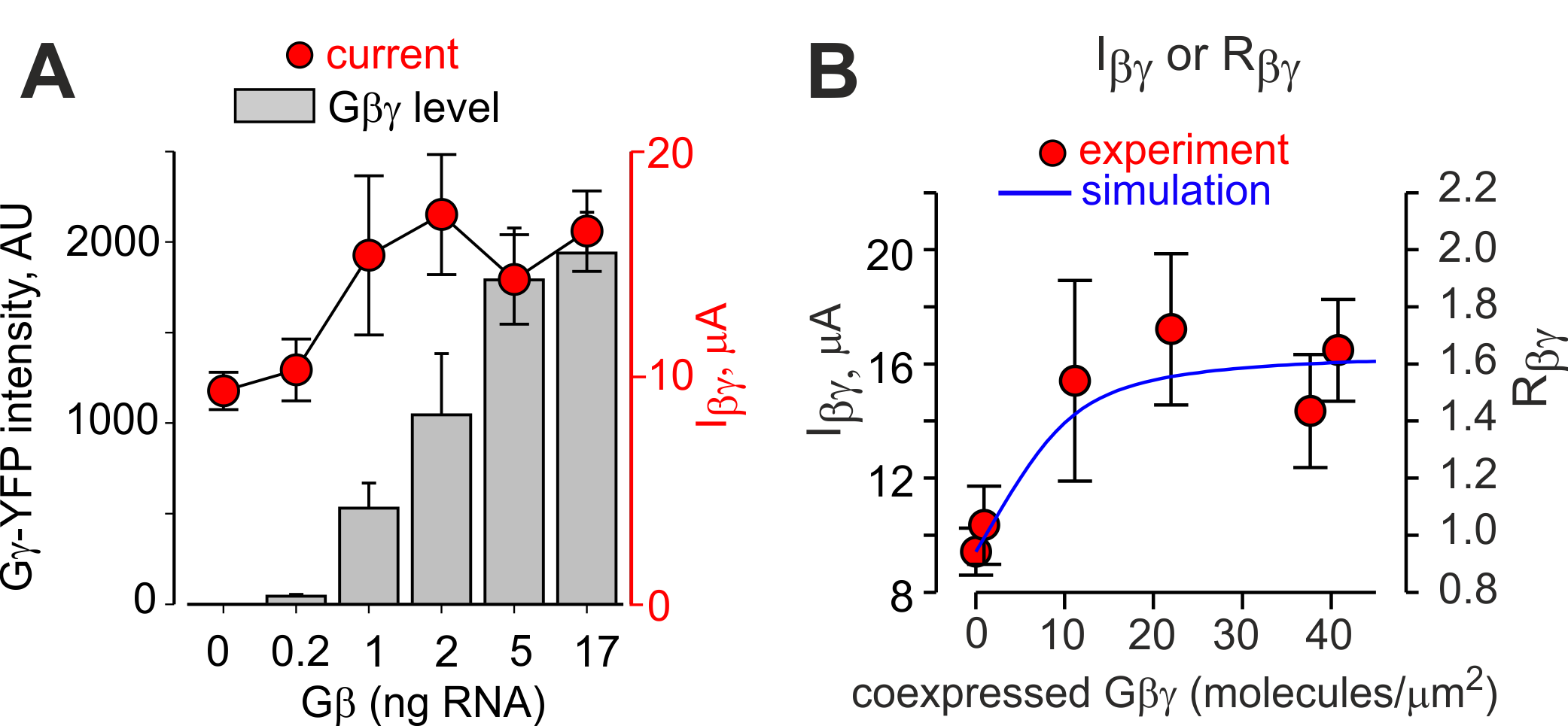

Supplement: S8 Fig — The presentation in similar to that of Fig 7. Gβ was coexpressed with Gγ-YFP in incremental doses, and with a constant amount (1 ng RNA) of wt GIRK1/2. RNA of Gγ-YFP was always half of that of Gβ RNA, by weight. (A) Gβγ-YFP fluorescence levels (grey bars, left Y-axis) and GIRK currents (red circles, right Y-axis) are shown on the same plot. GIRK1/2 density, calculated from Iβγ of the 17 ng Gβγ-YFP group, was 13 molecules/μm2. In addition, we injected YFP-GIRK1/GIRK2 (5 ng GIRK1-YFP) and measured Ibasal which was 8.4± 1.1 μA (n = 11), comparable to Ibasal of unlabeled GIRK1/2 (9.4±0.8 μA). Thus, we assumed the same density of ~13 channels/μm2 for labeled and unlabeled channels. Since the YFP-GIRK1/2 gave a fluorescent signal of 1237 ±221 AU (n = 7), this signal was assumed to correspond to 26 YFP molecules/μm2. This number was used as the basis of calculations of Gβγ-YFP density for plots shown in B. (B) Comparison of measured Iβγ or Rβγ (red circles) and simulated currents or Rβγ (blue curves). The left and right Y-axes are related to Iβγ and Rβγ, respectively. Available Gα and Gβγ (before Gβγ coexpression) were estimated from Itotal and Ibasal, giving 3.82 and 0.42 molecules/μm2 of Gβγ and Gα, respectively. (TIF) [file pcbi.1004598.s011.tif]

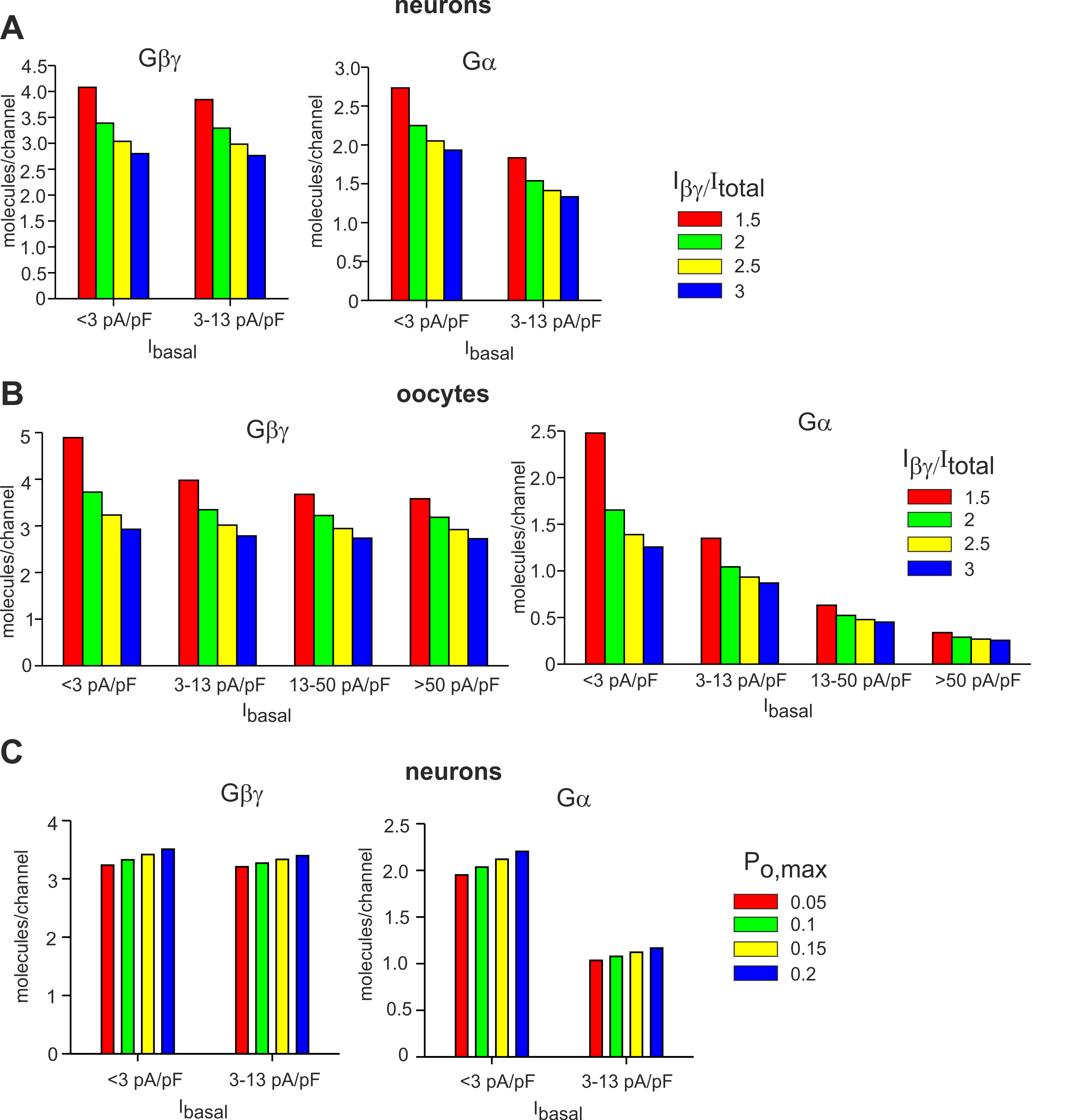

Supplement: S9 Fig — Whereas for the oocytes the actual Iβγ/Itotal ratio and Po,max are known, in neurons these parameters are not known. Both parameters affect the calculated channel density and could affect the estimates of stoichiometry. The calculations shown in this Figure demonstrate the same general trend in stoichiometries of GIRK1/2, Gβγ and Gα as we have found in the previous analysis in the oocytes, in a range of Iβγ/Itotal ratios (for neurons and oocytes; A and B) and Po,max (for neurons; C). The estimates of Gβγ are around 3-4/channel and relatively independent of Ibasal, and those of Gα are below 2 and drop sharply with the increase in Ibasal. Generally, the lowest channel density is most sensitive to perturbations, and, for the lowest simulated Iβγ/Itotal ratio, calculated Gβγ/channel and Gα/channel exceed our usual estimates. (TIF) [file pcbi.1004598.s012.tif]
